# Estimation of Global Network Statistics from Incomplete Data

**DOI:** 10.1371/journal.pone.0108471

**Published:** 2014-10-22

**Authors:** Catherine A. Bliss, Christopher M. Danforth, Peter Sheridan Dodds

**Affiliations:** Department of Mathematics and Statistics, Vermont Complex Systems Center, The Computational Story Lab, and the Vermont Advanced Computing Core, University of Vermont, Burlington, Vermont, United States of America; University of Warwick, United Kingdom

## Abstract

Complex networks underlie an enormous variety of social, biological, physical, and virtual systems. A profound complication for the science of complex networks is that in most cases, observing all nodes and all network interactions is impossible. Previous work addressing the impacts of partial network data is surprisingly limited, focuses primarily on missing nodes, and suggests that network statistics derived from subsampled data are not suitable estimators for the same network statistics describing the overall network topology. We generate scaling methods to predict true network statistics, including the degree distribution, from only partial knowledge of nodes, links, or weights. Our methods are transparent and do not assume a known generating process for the network, thus enabling prediction of network statistics for a wide variety of applications. We validate analytical results on four simulated network classes and empirical data sets of various sizes. We perform subsampling experiments by varying proportions of sampled data and demonstrate that our scaling methods can provide very good estimates of true network statistics while acknowledging limits. Lastly, we apply our techniques to a set of rich and evolving large-scale social networks, Twitter reply networks. Based on 100 million tweets, we use our scaling techniques to propose a statistical characterization of the Twitter Interactome from September 2008 to November 2008. Our treatment allows us to find support for Dunbar's hypothesis in detecting an upper threshold for the number of active social contacts that individuals maintain over the course of one week.

## Introduction

Data collected for complex networks is often incomplete due to covert interactions, measurement error, or constraints in sampling. Particular individuals may wish to remain hidden, such as members of organized crime, and individuals who are otherwise overt may have some interactions that they wish to remain hidden because those interactions are of a sensitive nature (e.g., romantic ties). In other instances, links may be erroneously inferred from spurious or noisy interactions. Furthermore, extremely large networks necessitate an understanding of how network statistics scale under various sampling regimes [1], [2]. Explorations of empirically studied networks have largely ignored these biases and consequently, characterizations of the observable (sub)networks have been reported as if they represent the “true” network of interest.

When members of a population are drawn at random, each with equal selection probability, the sample statistic being studied is often a good estimate of the population statistic. Problematically, subsampling networks often induces bias: some individuals or interactions may be more likely to be selected [3]. Consider, for example, a network for which a random selection of links is observed. The collection of observed nodes in such a subnetwork is biased because large degree nodes are more likely to be included in the sample than nodes of small degree.

The development of techniques to correct sample estimates of population statistics is needed to enable more accurate portrayals of empirically studied large -scale networks and aid in efforts to model dynamics such as cascading failures and complex contagion [4]–[7].

A central confounding issue is that the errors introduced by biases in sampling may be exacerbated both by particular sampling strategies and by various underlying network topologies of the true network from which the subsamples are chosen [8]–[15]. Researchers have explored the effects of sampling by nodes [1], [9], [13], [16]–[18]; sampling by edges or messages [1], [2], [18]; and graph exploration methods based on random walks, snowball sampling, and respondent driven sampling [1], [19], [20].

We organize our paper as follows. First, we outline some of the most common global network statistics. In the Methods and Materials section, we describe our data and sampling strategies. In the Analysis section, we describe scaling methods for global network statistics and apply our methods to four classes of simulated networks and six empirical datasets. We provide a summary of all our estimates in Table 1. In the subsequent section, we apply our methods to Twitter reply networks as both a case of scientific interest and demonstration of our methods. In the Discussion, we discuss the implications of our findings and suggest further areas of research.

**Table 1:** Summary of scaling techniques.

	Sampled	Failed	Sampled	Sampled
	nodes	links	links	interactions
Predicted number of nodes ( 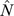 )	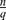	*n*	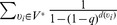	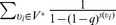
Predicted number of edges ( 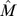 )	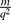	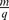	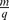	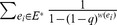
Predicted average degree 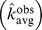	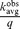	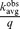	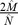	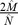
Predicted clustering ( 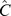 )	*C*	*qC*	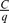	–
Predicted max. degree 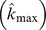	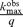	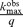	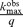	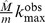

### Global network statistics

Real complex networks have come to be characterized by a range of functional network statistics. In this paper, we explore how descriptive measures such as the

- the number of nodes, *N*,
- the number of edges, *M*,
- degree distribution, *P_k_*,
- the average degree, *k*_avg_,
- the max degree, *k*_max_,
- clustering coefficient, *C*, [21], and
- the proportion of nodes in the giant component, *S*,

scale with respect to missing network data. Based on our observations, we suggest predictor methods for inferring these network statistics from subsampled network data.

The most important structural feature of a network is the degree distribution, *P_k_*, and this has been the focus of much previous work on subsampled networks. The classical Erdös-Rényi random graph model famously exhibits a Poisson degree distribution, 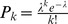 [22]. In contrast to Erdös-Rényi random networks, preferential attachment growth models describe a random process whereby new nodes attach with greater likelihood to nodes of large degree giving rise to a Power-law or Scale-free degree distribution, 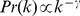 [23]–[26]. Other distributions, such as lognormals and power-laws with exponential cutoffs may equally characterize the degree distributions of some empirical networks [27].

Previous work has explored how the degree distribution is distorted when the subnetwork is the induced subgraph on sampled nodes [9], [10], [13], [17], [18], [28]–[30]. Han et al. [9] investigated the effect of sampling on four types of simulated networks: random graphs with (1) Poisson, (2) Exponential, (3) Power-law, and (4) Truncated normal distributions. They observed that degree distributions of sampled Erdös-Rényi random graphs appear to be linear on a log-log plot. Others have also suggested that subnetworks of Erdös-Rényi random graphs appear “power-law-like” and could be mistaken for a scale-free network [9], [17]. Typically, scale-free networks have degree distributions which span several orders of magnitude and thus, subnetworks of Erdös-Rényi random graphs would not be classified as scale-free networks by most researchers. As warned in [27], further errors may be incurred when attempting to use linear regression to fit a power-law.

Stumpf and Wiuf [28] examined how degree distributions of Erdös-Rényi random graphs scale when subnetworks are obtained through uniform random sampling on nodes and “preferential sampling of nodes,” whereby large degree nodes have a greater probability of being selected. They showed that Erdös-Rényi random graphs exhibit closure under subsampling by nodes (i.e., an Erdös-Rényi random graph sampled by nodes is again an Erdös-Rényi random graph). Erdös-Rényi random graphs did not exhibit closure under preferential sampling of nodes.

Stumpf et al. [13] suggested that the degree distribution of the subnetwork induced on randomly selecting nodes is independent of the proportion of nodes sampled and that the true degree distribution can only be determined by knowledge of the generating mechanism for the network. Unfortunately, this is often not known or fully understood.

Several researchers have explored techniques for estimating the true degree distribution from subnetwork data. We first examine the subnetwork degree distribution before examining attempts to solve for the true degree distribution in terms of the subnetwork degree distribution. We consider three cases. First, when links are sampled with probability *q* and the subnetwork is taken to be the network generated on sampled links, the probability that a node of degree *i* in the true network will become a node of degree *k* in the subnetwork (*k*≤*i*) is given by 
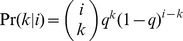
. The subnetwork degree distribution can be determined by weighting these probabilities by *P_i_*, the probability of node *i* appearing in the true network [31]. The subnetwork degree distribution is then given by 
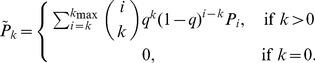
(1)


Next, we consider subnetworks obtained by link failure. In these cases, all nodes are observed, only a proportion (*q*) of links are observed. This cases is nearly identical to Equation 1, except for the presence of nodes of degree zero. 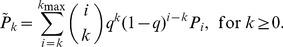
(2)


Lastly, we consider subnetworks obtained from the induced network on sampled nodes. In this case, the probability of observing a node is *q*. As such, 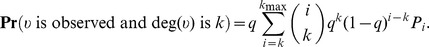



We note that this is not the observed subnetwork degree distribution because when a subnetwork obtained from the induced network on sampled nodes is observed, the frequencies of nodes of degree *k* are computed relative to the number of observed nodes. This becomes 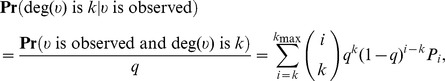
which is normalized. For added clarity, consider a network of *N* nodes and *M* = 0 edges. We observe that 
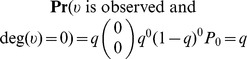
 whereas 
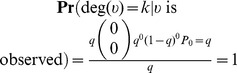
. The latter agrees with our observation, namely the (observed) network induced on sampled nodes will have all nodes of degree 0 and an observed probability distribution which is simply *P*_0_ = 1.

Viewing Equation (1) as a system of *k* equations, we may derive an expression for the true degree distribution in terms of the observed subnetwork degree distribution. We refer the interested reader to Materials S1 for the derivation of this result:

Given a network with degree distribution *P_j_*, with sampling fraction *q*, and the subnetwork degree distribution 
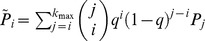
, we may solve for *P_j_* in terms of the subnetwork degree distribution 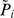. This yields 
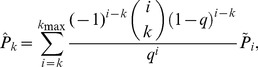
(3)where 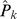 represents the predicted degree distribution and nodes of degree 0 are handled appropriately.

Verification of this result is also presented in Materials S1.

Our derivation differs from Frank [29] by a factor of 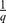, 
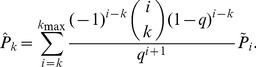
(4)


Equation 4 solves 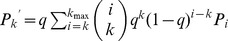, for *P_i_* in terms of 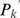, however 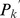 is not the observed degree distribution. Neither of these derivations, however, are guaranteed to be non-negative [3] and their practicality of use is limited.

Model selection methods provide a different approach by employing maximum likelihood estimates to identify which type of degree distribution characterizes a true network, given only a subnetwork degree distribution [32]. Although these methods are able to discern that some network degree distributions may be better characterized by lognormal or exponential cutoff models instead of power-laws, only models selected *a priori* for testing form the candidate pool of possible distributions.

In contrast to the model selection technique proposed by Stumpf et al. [32], we explore a probabilistic approach which utilizes knowledge of the proportion of sampled network data (*q*) and the subnetwork degree distribution. In doing so, we desire an estimation that captures the qualitative nature of the degree distribution without making any assumptions about candidate models. We show that reasonably good estimates of *P_k_* can be achieved with no knowledge of the generating mechanism. With a reasonable estimate of the degree distribution available, we are able to overcome a previously noted obstacle identified by Kolaczyk [3] who notes that predictors for network statistics (sampled by links) have proven more elusive because of the need for knowledge of the true degree distribution [3]. Our method can be used in conjunction with Hortiz-Thompson estimators to reasonably predict network statistics for cases where node selection is not uniform (i.e., subnetworks generated by sampled links or weights).

In the subsequent sections, we summarize this work and show how our method surmounts this obstacle. To our knowledge, scaling techniques for networks generated by sampled interactions (e.g., weighted networks) have not been addressed in the literature and given the interest in large, social networks derived from weighted, directed interactions, we find this analysis timely and relevant.

## Materials and Methods

In this paper, we focus on four sampling regimes: (1) subnetworks induced on randomly selected nodes, (2) subnetworks obtained by random failure of links, (3) subneworks generated by randomly selected links, and (4) weighted subnetworks generated by randomly selecting interactions. Motivated by our work with Twitter reply networks [33] for which we have a very good approximation of the percent of messages which are obtained, we base our work on the assumption that the proportion of missing data is known. This is a critical assumption and one that we acknowledge may not always be satisfied in practice. Efforts to estimate the proportion of missing nodes or links are intriguing, but are beyond the scope of this paper.

### Unweighted, undirected networks

Our data consist of simulated and empirical networks. We generate unweighted, undirected networks with *N* = 2×10^5^ nodes and average degree *k*_avg_ = 10 according to four known topologies: Erdös-Rényi random graphs with a Poisson degree distribution [22], Scale-Free random graphs with a power-law degree distribution [24], [34], Small world networks [35], and Range dependent networks [36]. Erdös-Rényi, Scale-free, Small world, and Range dependent models were constructed with the CONTEST Toolbox for Matlab [37]. We note that the small world networks were set to have random rewiring probability *p* = 0.1 and preferential attachment networks were set to have *d* = 5 new links when they enter the network. Range dependent networks were set to establish a link between nodes 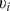 and 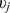 with probability 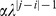 where we set 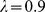 and 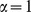. As noted by [37], this choice of *α* ensures that nodes 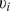 and 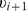 are adjacent and 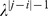 ensures that short range connections are more probable than long range connections. We also examine six well known empirical network datasets: *C. elegans* [35], [38], Airlines [39], Karate Club [40], Dolphins [41], Condensed matter [42], and Powergrid [35].

**Figure 1:**
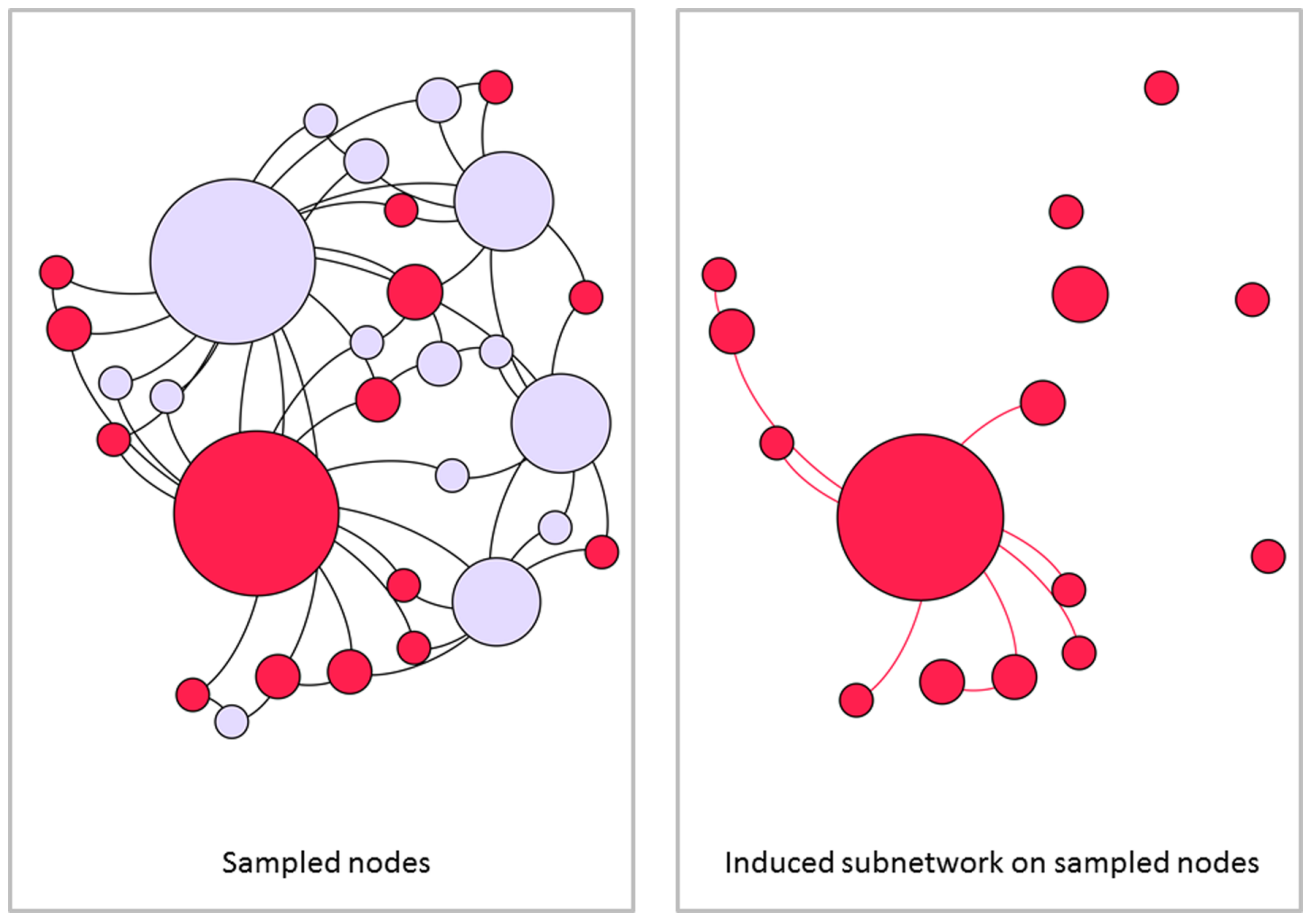
Node induced subnetwork on randomly sampled nodes. (Left) The true network is sampled by randomly selecting nodes (red). (Right) The node induced subnetwork consists of sampled nodes and edges whose endpoints both lie in the collection of sampled nodes.

**Figure 2:**
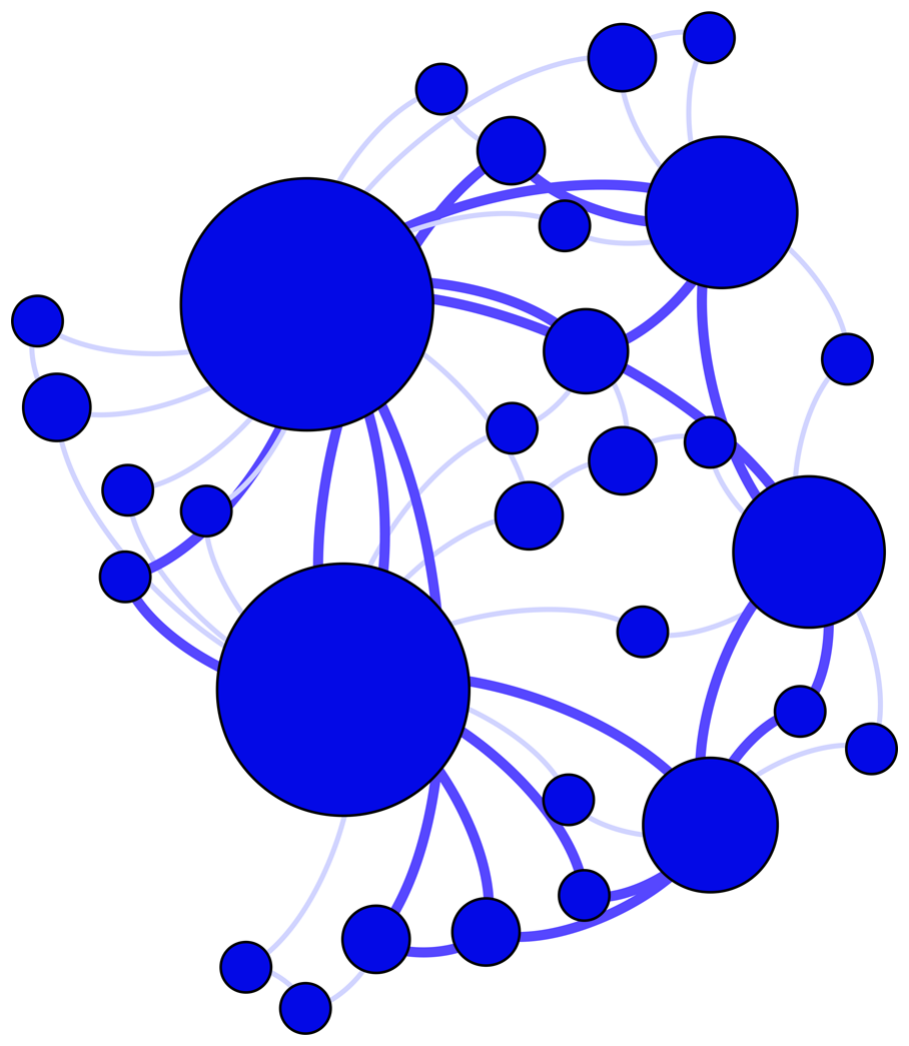
Failed link subnetwork. Hidden or missing links are depicted in grey. All nodes remain in the subnetwork and only visible or sampled links remain.

**Figure 3:**
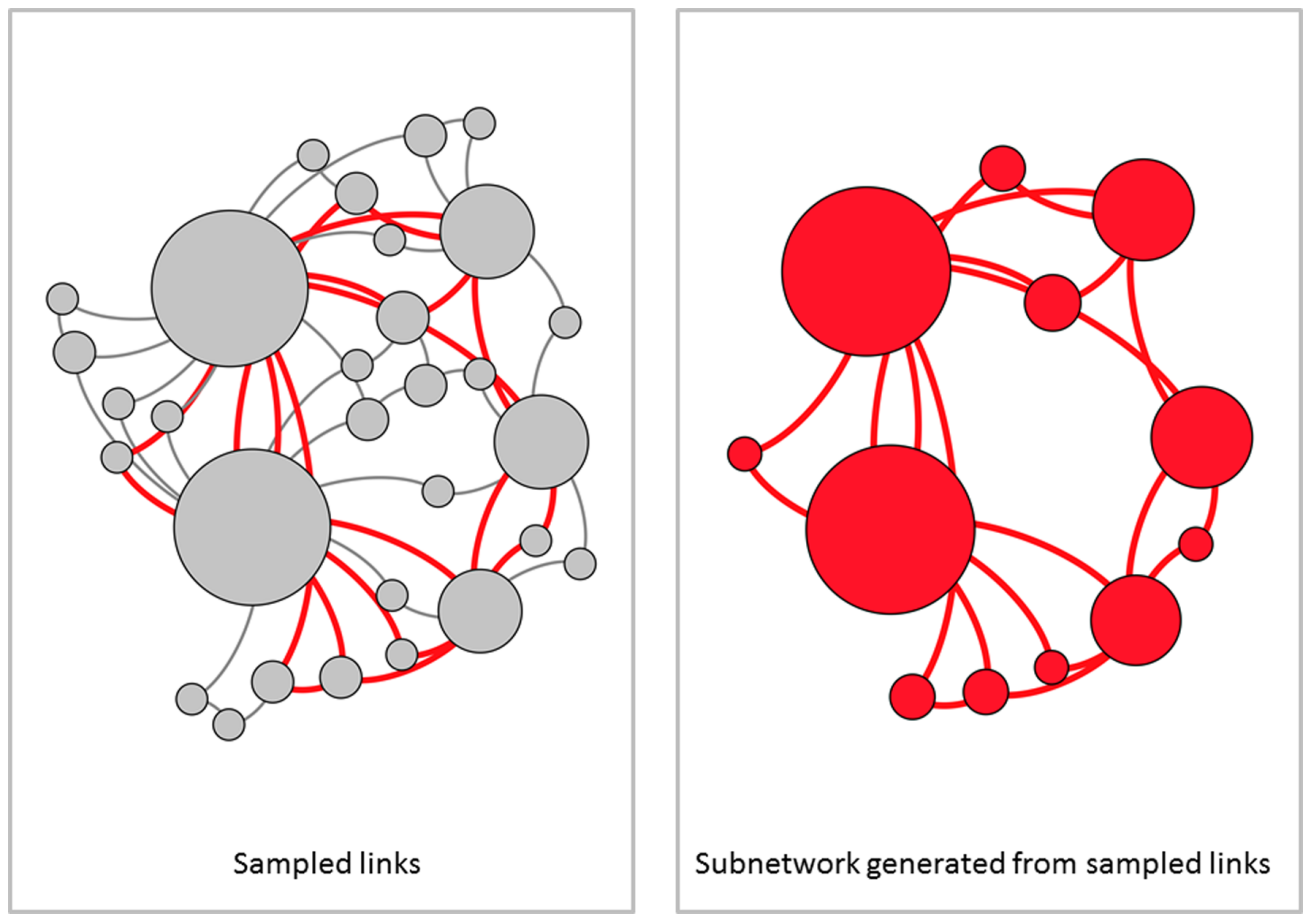
Subnetwork generated from sampled links. (Left) A network is sampled by randomly selecting links shown in red. (Right) The subnetwork consists of all sampled links and only nodes which are incident with the sampled links. In this type of sampling, no nodes of degree zero are included in the network. Large degree nodes are more likely to be included in the subnetwork.

We sample each of these simulated and empirical networks and examine the subnetwork induced on sampled nodes (Fig. 1), the subnetwork obtained by failing links (Fig. 2), and the subnetwork generated by sampled links (Fig. 3). For a given network, 100 simulated subnetworks are obtained for a given sampling strategy and subsampling percentage *q*, as *q* varies from 5% to 100% in increments of 5%.

### Weighted, undirected networks

We examine the effects of uniformly increasing edge weight (Experiment 1, Cases I–V) as well as the distribution of edge weights (Experiment 2, Cases VI and VII) on the scaling of network statistics (Table 2).

**Table 2:** Summary of weighted network experiments.

Case	*k*_avg_	*w*_avg_	Distribution of weights
I	6	1.0	*w*(*e_j_*) = *w*_avg_ (uniform)
II	6	2.0	*w*(*e_j_*) = *w*_avg_ (uniform)
III	6	3.0	*w*(*e_j_*) = *w*_avg_ (uniform)
IV	6	4.0	*w*(*e_j_*) = *w*_avg_ (uniform)
V	6	5.0	*w*(*e_j_*) = *w*_avg_ (uniform)
VI	6	5.0	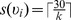 (equal effort)
VII	6	5.0	*w*(*e_j_*) = *randi*{1..9} (randomized)

Note: *w*(*e_j_*) refers to the weight of edge *e_j_*, 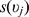 refers to the strength of node 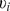) and *randi*{1..9} refers to a randomly selected integers between 1 and 9 (inclusive).

#### Experiment 1: Uniform distribution of edge weights

**Figure 4:**
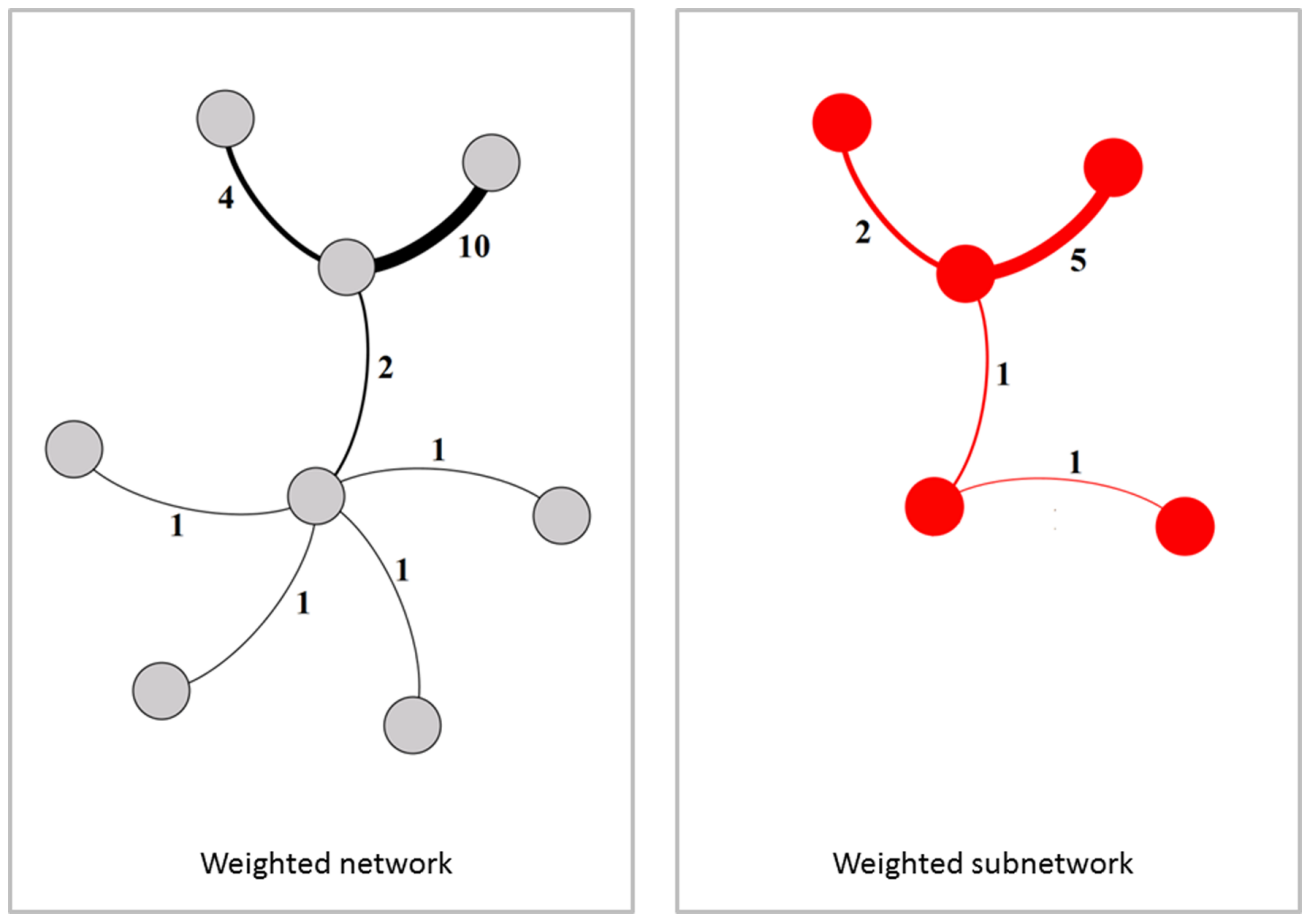
Weighted subnetwork generated from sampled interactions. (Left) An unsampled weighted network consists of nodes, links and weights representing the number of interactions represented by the link. (Right) Sampling by interacting produces a subsample whereby links are included in the subsample only if at least one interaction has been sampled. The subnetwork is the induced subgraph on these links with *w_i_*≥1.

In this set of experiments, we generate Erdös-Rényi networks with *N* = 2000 nodes and *k*_avg_ = 6. We assign each edge to have equal weight, *w*, where *w* = 1, 2, 3, 4, or 5 (corresponding to Cases I–V). We similarly generate Scale-free networks with *N* = 2000 nodes and *k*_avg_ = 6. We then sample each of the weighted, undirected networks by randomly selecting 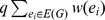 interactions and examine the subnetwork generated by links with *w*(*e_j_*)>0 (Fig. 4). This procedure is repeated to generate one hundred simulated networks for each class and varying proportions of sampled interactions (*q*).

#### Experiment 2: Non-uniform distribution of edge weights

In this set of experiments, we explore how the distribution of weights on edges can impact scaling of global network statistics. As in the previous case, we first generate an Erdös-Rényi network with *N* = 2000 and *k*_avg_ = 6. We then add weights to edges in one of two ways. In Case VI, we assume “equal effort” in that all nodes will have an equal number of interactions distributed equally among their incident edges. This requirement ensures that all nodes have equal node strength and that effort is equally distributed to each neighbor. More specifically, for node 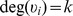, we set each of the *k* edges to have weight 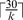. In Case VII, for each edge we select an integer weight between 1 and 9 from a uniform probability distribution. Certainly, other variants of the weight distribution exist and their analysis may provide additional insight in future studies.

### Weighted, directed networks–Twitter reply networks

Twitter reply networks [33] are weighted, directed networks constructed by establishing a directed edge between two individuals if we have a directed reply from a individual to another during the week under analysis. These networks are derived from over 100 million tweets obtained from the Twitter streaming API service during September 2008 to February 2009. We refer the interested reader to [33] for more information. The data for these networks is provided at http://www.uvm.edu/storylab/share/papers/bliss2014a/. During this time, we obtained between 25% to 55% of all tweets (Table S24 in Materials S1). Using the scaling methods developed in the Estimating global network statistics section, we predict global network statistics for the Twitter interactome during this period of time by viewing in- and out-network statistics separately (e.g., two distinct networks) to account for directionality.

## Analysis

### Sampling by nodes

Given a network, *G* = (*V*, *E*), where *V* is the collection of nodes (or vertices) and *E* is the collection of links (or edges), we randomly select a portion of nodes *q*, where 0<*q*≤1. The node induced subgraph on these randomly sampled nodes is given by *G* ^*^ = (*V* ^*^, *E* ^*^), where *V* ^*^ represents the randomly selected nodes and *E* ^*^ represents the edges in *E* for whom both endpoints lie in *V* ^*^ (Fig. 1). This type of sampling occurs when a selected group, representative of the whole, is observed and all interactions between sampled individuals are known. This sampling strategy is well studied and we will only view key results here (see [3]).

### Scaling of *N*, *M*, *k*_avg_, *C*, *k*_max_, and *S*

Given a subnetwork of size *n* = *qN* known to be obtained by randomly selecting *qN* nodes, the number of nodes in the subsample clearly scales linearly with *q* (see Figs. S1a and S2a in Materials S1). The size of the true network is predicted by 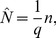
(5)which shows good agreement with the true network statistic (Table S1 in Materials S1). Note that this result is independent of network type and is only dependent on *q*, the fraction of nodes subsampled, and *n*, the size of the subsample.

Given a network with *N* nodes and a subnetwork of *n* nodes, the probability of selecting edge *e_ij_* is given by 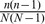. This is simply the probability that the two nodes, 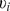 and 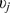, incident with the edge *e_ij_*, are selected. The number of edges in the subnetwork is found by 
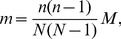
(6)where *m* represents the number of edges in the subnetwork and *M* represents the number of edges in the true network. For large networks, *m*≈*q* ^2^ *M*. This agrees well with simulated results (Figs. S1b and S2b in Materials S1). The predicted number of edges is given by 
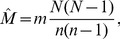
(7)which scales as 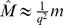 for large networks. This predictor shows good agreement with actual values (Table S2 in Materials S1).

The average degree, *k*_avg_, is found by 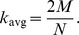



Given expressions for the expected number of edges (7) and the expected number of nodes (5), the expected average degree of a true network, 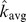, based on an observed average degree of a subnetwork:
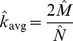
(8)

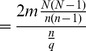
(9)

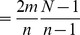
(10)

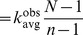
(11)

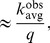
(12)where in line (10) we have assumed that 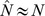, 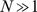 and 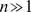. Comparing this result to simulated subnetworks induced by subsampling nodes (Figs. S1c and S2c in Materials S1), we find very good agreement between the predicted average degree and true average degree (Table S3 in Materials S1), except for the small empirical networks (Karate club and Dolphins) sampled with low *q*. In these cases, we violate the assumption that 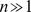 because subsamples of the Karate Club network degenerate to subnetworks of 3 edges or less when *q*≤0.20. Similarly, subsamples of the Dolphin network degenerate to subnetworks of 3 edges or less when *q*≤0.15. When the observed number of edges in the subnetwork exceeds 3, our predicted 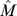 has an error less than 5% (Table S3 in Materials S1).

The scaling of the max degree is highly dependent on network type, or more precisely, the relative frequency of high degree nodes. For networks with relatively few large hubs and many small nodes of small degree, *k*_max_ scales linearly with *q* and 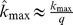. For networks with many nodes of maximal degree *k*_max_ scales nonlinearly with *q* (Figs. S1d and S2d in Materials S1). An example of this would be a regular lattice. All nodes have the same (and hence maximal) degree. This pathological example is not often seen in practice. Simulated Small world networks begin as a regular lattice with random rewiring probability, *p*. Since our Small world networks have *p* = 0.1, our Small world networks exhibit this pathological behavior more so than several empirical Small world networks. We note that this is simply a matter of tuning *p* and not indicative of all Small world networks.

This distinction makes predicting the maximum degree more challenging since an accurate predictor ultimately relies on knowledge of the network type - knowledge one usually does not have in an empirical setting. Our proposed technique utilizes 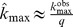, unless our algorithm detects a large number of nodes with degree similar to *k*_max_ and are assured that the subnetwork that has not degenerated to a small network (*n*<30). More specifically, if our algorithm detects 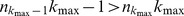, then we use the adjustment Equation 13, where 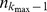 represents the number of nodes of degree *k*_max_−1. In this case, 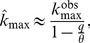
(13)where *θ* = the number of nodes with degree greater than 75% of *k*_max_.

The rationale for this rough approximation is that the nodes which have high degree (>75% of the observed max. degree) may have been nearly equal contenders for losing a neighbor during subsampling. When all nodes have equal degree, the denominator of Equation 13 tends to 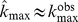. Table S4 in Materials S1 presents the error for this predictor and demonstrates that our method performs reasonably well for most networks in our data set. To our knowledge, this is the first attempt to characterize how *k*_max_ scales with subsampling and we hope that future work improves upon our estimate.

We measure clustering using Newman's global clustering coefficient [21] 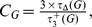 where 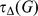 denote the number of triangles on a graph and 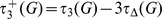, which is the number of vertex triples connected by exactly two edges (as in the notation used by [3]). Since the probability of selecting a node is *q*, both the number of triangles and connected vertex triples scale as *q* ^3^. Thus, 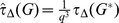 and 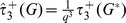 [43]. We then expect 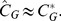
(14)


This is supported by simulations (Figs. S1e and S2e in Materials S1) and small errors in 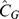 (Table S5 in Materials S1). We note that for small *q*, some subnetworks completely breakdown and no connected triples are present. In these situations, the clustering coefficient can not be computed nor can the true network's clustering coefficient be well predicted.

We next explore how the size of the giant component scales with the proportion of nodes sampled (Fig. S1f and S2f in Materials S1). For the Erdös-Rényi and Scale-free random graphs, the giant component emerges when the subnetwork has 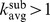. This occurs when *qk*_avg_>1 and so for our simulated Erdös-Rényi and Scale-free networks, this occurs when *q* = 0.10 because the true networks have *k*_avg_ = 10. The thresholds for the emergence of the giant component in Small World and Range dependent networks are much higher. This may be due to the relatively large clustering coefficients of these networks. As suggested by Holme et al. [44], networks with a large clustering coefficient [35] are more vulnerable to random removal of nodes. We observe the same trend with Newman's global clustering coefficient.

In the case of the empirical networks, we find that the giant component emerges for *q* corresponding to 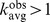. *C. elegans*, Airlines, and Condensed Matter networks are more resilient to random removal of nodes in that the giant component persists for small levels of *q*. This is most likely due to their relatively high average degrees, as compared to the other networks (heterogeneity of nodes' degrees in these networks). Heterogeneous networks demonstrate more resilience due to random removal of nodes at high levels of damage [45]. In general, it may be very difficult to predict the exact critical point at which the giant component emerges from subnetwork datasets.

#### Scaling of *P_k_*

The complementary cumulative degree distribution (CCDF) becomes more distorted as smaller proportions of nodes are sampled, as shown in Figure S3 in Materials S1 and given by Equation 1. Subnetworks obtained by the induced graph on sampled nodes will often have 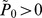. This occurs when 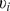 is selected in sampling, but no neighbors of 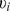 are selected in the sample.

Our goal is to predict the degree distribution, given only knowledge of the proportion of nodes sampled (*q*) and the subnet degree distribution. We note that the probability that an observed node of degree *k* came from a node of degree *j*≥*k* in the true network is given by 
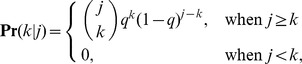
where *q* is the probability that a node's neighbor was included in the subsample and 1–*q* is the probability that a node's neighbor is not included in the subsample.

After normalizing, we find 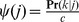 describes the normalized probability that an observed node of degree 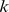 came from a node of degree 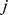 in the true network, where 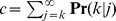. Note that when 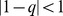 this series converges and we find 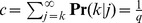. Thus, 
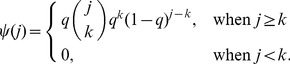
(15)


Let 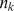 represent the number of nodes of degree *k*. We compute 
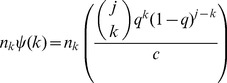
(16)

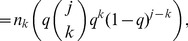
(17)where we use Stirling's approximation to estimate the binomial coefficients for large *j*. We have taken care to include observed nodes of degree zero in this process (e.g., 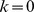 in Equation 16).

For networks with nodes of large degree (e.g., hubs), one can further speed up the computation and reduce floating point arithmetic errors by mapping back observed nodes of degree *k* to the expected value of the distribution obtained in Equation 15: 
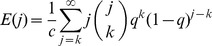
(18)

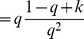
(19)

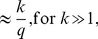
(20)where 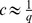. In making use of 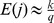, we perform a separate calculation for nodes of degree zero: 
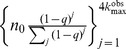
. In all cases, we assume a finite network. We limit our calculations to 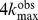 as a rough estimate on the upper bound needed for the sum in Equation 15.

Figure S4 in Materials S1 reveals the predicted degree distribution for subnets induced on varying levels of randomly selected nodes. To test the goodness of fit for the estimated degree distribution and the true *P_k_*, we apply the two sample Kolmogorov-Smirnov test. Figure S16 in Materials S1 shows the *D* test statistics for the predicted degree distributions for both estimation methods (Equations 16 and 18), as well as the *D*_crit_ computed from 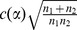, where 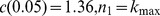 and 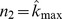. For most networks, *D*≤*D*_crit_ for *q*≥0.3, suggesting that when at least 30% of network nodes are sampled, our methods provide an estimated degree distribution which is statistically indistinguishable from the true degree distribution. Although we reject the null hypothesis for the preferential attachment case, for all 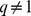, we wish to point out the potential for bias in the Kolmogorov-Smirnov test with large *n* [46]. As shown, *D*_crit_ values are quite low and the bias in this test is due to large *n*_1_ and *n*_2_. The statistical power in this test leads to the detection of statistically significant differences, even when the absolute difference is negligible. Thus, we caution the interpretation of this statistical test and place more interest in the value 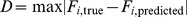, where 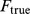 and 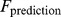 represent the true and predicted CDFs.

### Link failure

We now turn our attention to link failure. As in the previous cases, we denote the true, unsampled network as 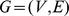. Some proportion, *q* of links remain “on” (or present in the sample) and 1–*q* are hidden or undetected by sampling. 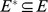 consists of precisely the links that remain “on” and 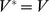 (Fig. 2). Figures S5–S6 demonstrate how network statistics scale in this sampling regime.

In this case we may use the estimator to predict the number of nodes, 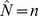 and we may predict the number of edges by 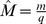. The average degree is found by 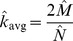
(21)

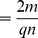
(22)

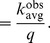
(23)


Using Newman's global clustering coefficient 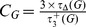 [21], we note that 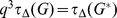 and 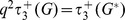 because each edge is selected with probability *q*. Thus, 
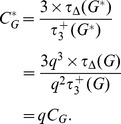



Thus, 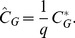
(24)


We compute the maximum degree with the same method as described in in the subsection on sampling by nodes because the number of neighbors of a node scales the same in both cases. Using these estimates, we find relatively low error in the predicted the network measures for 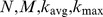, and 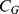 (Tables S6–S10 in Materials S1).

Several networks' giant components exhibit similar patterns of resilience when sampling by nodes or failing links. Comparing the resilience of the proportion of nodes in the giant component under sampling by nodes vs. failing links, we see that Erdös-Rényi random graphs, random graphs with preferential attachment, Airlines, Condensed matter, *C. elegans*, and Powergrid networks all perform relatively similarly under the two sampling regimes. A noticeable difference is seen in Small world, Range dependent, Karate club, and Dolphin networks. In the case of Small world and Range dependent networks, the regularity of the underlying lattice in these networks means that each time a node is not observed, this also means that *k*_avg_ edges are also missing. Given that the majority of nodes have roughly the same degree for these networks, subsampling fractures the giant component quickly (i.e., for *q* around 0.7 and 0.8 respectively). In the case of the small Karate club and Dolphins networks sampled by nodes, the proportion of nodes in the giant component increases with decreasing *q*. In these cases, the network consists of relatively few nodes, which are connected. In contrast, when examining the failing links case, we have all nodes present, but these nodes are missing almost all links and the network is highly disconnected.

Figure S7 in Materials S1 reveals the distortion of the CCDF when links fail in a network and all nodes remain known to the observer. Clearly, there are nodes of degree zero that are observed in this sampling regime. The predicted degree distribution is obtained by the methods described under sampling by nodes (including the treatment of observed nodes of degree zero) and presented in Fig. S8. The results of the two sample Kolmogorov-Smirnov test reveal that the estimated degree distribution and the true degree distribution are statistically indistinguishable for *q*≥0.3 for most networks (Fig. S17 in Materials S1). As previously noted, the large number of observations in degree distribution for the random graph grown with preferential attachment leads to high statistical power and a low *D*_crit_.

### Sampling by links

The problem of missing links may also manifest itself in another manner. In contrast to the case when all nodes are known and some links are hidden, we now consider subnetworks generated by sampled links and the nodes incident to those links (Fig. 3). This type of sampling occurs in many social network settings, such as networks constructed from sampled email exchanges or message board posts. In this case, we have data pertaining to messages (links). Nodes (individuals) are only discovered when a link (email) which connects to them is detected.

In this case, edges are sampled uniformly at random and we may use our previous estimator, 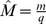. Node inclusion is biased, however, in that nodes of high degree will be detected with greater probability than nodes of low degree precisely because they are more likely to have an incident edge sampled.

To motivate an appropriate predictor, we must first consider how the number of nodes in a subnetwork obtained by the subnetwork generated by sampled links scales with *q* (Figs. S9a and S10a in Materials S1). To do this, let us consider the probability that a node is included in such a subsample. If the number of edges not sampled (*M*–*m*) is less than the degree 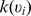 of node 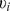, then we can be certain that our node of interest will be detected in sampling. On the other hand, if 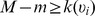, then the probability of 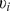 being in the subnetwork scales nonlinearly with *q*. Using the framework set forth by Kolaczyk [3], observe that there are 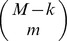 ways of choosing *m* edges from the 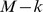 edges not incident with node 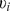 and there are 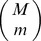 total ways of choosing *m* edges from all *M*. Thus, we have 
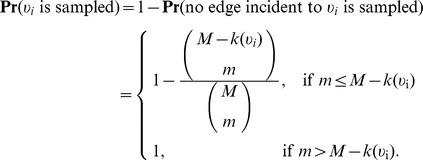



The Horvitz-Thompson estimator given by 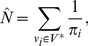
(25)where 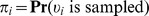.

Kolaczyk [3] warns that this may not be a useful result, due to the fact that the true degree of a given node is likely to be unknown. We overcome this limitation by using our predicted degree distributions obtained by the techniques previously mentioned. Observe that when sampling by links, no nodes of degree zero will be observed. We also note that in the case when 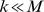 and *m*, we may make the following approximation which is less computationally burdensome: 
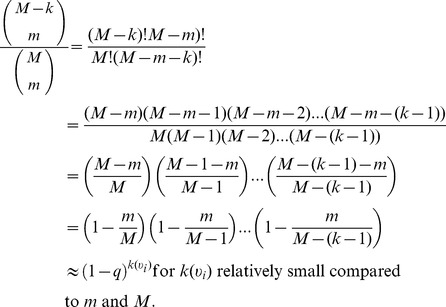



This is simply the probability that a node of degree 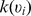 loses all edges during subsampling 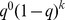 and thus 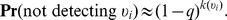 Thus, 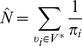
(26)

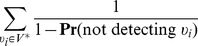
(27)

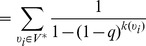
(28)


We apply these methods to our simulated and empirical networks.

Once 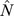 and 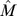 have been computed, the average degree is simply 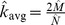. The max degree scales roughly linearly for preferential attachment models and many of the empirical networks, however scales sublinearly in networks with a high proportion of nodes of similar degree (e.g. the regular lattice structure seen in Small world and Range dependent networks). Clustering scales approximately as 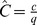 and the giant component shows a critical threshold which varies according to network type and average degree. The relative errors of our predictors are summarized in Tables S11–S15 in Materials S1. The scaling of 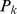 and the predicted degree distribution are presented in Figs. S11 and S12.

To test the goodness of fit for the estimated degree distribution and the true 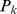, we again compute 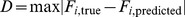, two sample Kolmogorov-Smirnov test statistic (Fig. S18 in Materials S1). This figure shows that reasonable results are achieved when 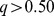, a noticeable increase in the percent of network knowledge needed, as compared to other sampling strategies (sampling by nodes and failing links).

### Sampling by interactions

Lastly, we consider the case of sampling by interactions in the special case of a weighted network (Fig. 4). In this case, we begin with 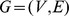, where *E* is a set of edges, *e_j_*, with weight 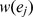. The weight on an edge represents the number of interactions between two vertices. An alternative representation is simply a network with multiple edge between two such vertices, one for each interaction. A subnetwork generated by 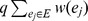 sampled interactions is simply a sampled collection of multi-edges and the nodes incident to these edges (e.g., the subnetwork generated by links with nonzero weight and nodes incident to those edges).

To consider how the number of nodes scales, we consider a similar formulation as discussed in the previous section for the probability that a given node is selected when sampling by links, however instead of the degree of a node, 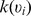, we are now interested in the strength of a node. The strength of a node is given by 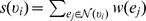, where 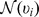 denotes the neighborhood of vertex 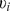 [47]. Let 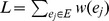 represent network load and 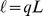, the number of sampled interactions. If the number of interactions which are not sampled (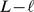) is less than the strength of a node 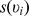, then we can be certain that node 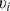 will be detected in sampling.

On the other hand, if 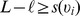, then there are at most 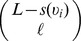 ways of choosing 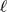 interactions from the 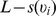 interactions not involving node 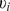. As an upper bound, we assume that the 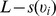 interactions are distributed over 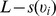 edges (weight of 1 on each edge) which maximizes the number of ways these could be chosen. There are at most 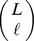 total ways of choosing 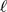 (distinct, labeled) interactions from all 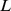. Letting 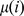 represent the probability that 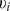 is sampled, we have 
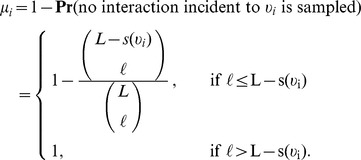



Thus, our Horvitz-Thompson estimator is, 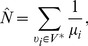
(29)where 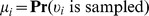. This can be well approximated by 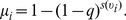
(30)


It should be noted that the strength of a node is merely predicted. Thus, effort must be made to predict the node strength distribution in the same spirit as was previously done for the degree distribution. To predict the node strength distribution, we modify Equation 17 and predict an observed node of strength 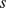 to be of strength 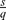 in the true network. Applying this corrector to our subsampled weighted networks, we find low relative error in the predicted number of nodes for most networks (Tables S16 and S17 in Materials S1). An exception to this is Case I (Erdös-Rényi) for *q*<0.55. We predict the node strength to be 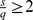 and yet in this case, the true network is unweighted (e.g., 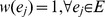). If there is knowledge that the network is unweighted, this example shows that the techniques from sampling by edges subsection will yield much better results.

We now consider how the number of edges in the subnetwork scales with the proportion of sampled interactions. The probability of selecting an edge 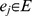 is equal to 1-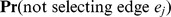. Notice that when the 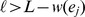, the edge 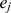 is certain to be included in the subsample. When 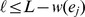, the probability of not selecting edge 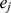 is simply the number of ways of selecting the 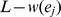 interactions 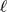 at a time, which are not on edge 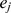 divided by the number of ways of selecting 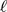 weights from 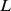. 
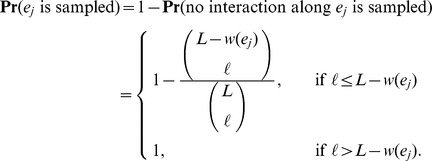



Thus, our Horvitz-Thompson estimator is, 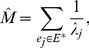
(31)where 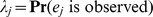, which is well approximated by 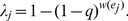
(32)


Again, we must have knowledge of the edge weights, or be able to predict them with reasonable accuracy. To do this, we predict an edge of weight 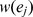 in the subnetwork to be of edge weight 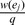 in the true network.

As the weights on edges tends to 1 (the unweighted network case), we retrieve our result for how edges scale when links are sampled (synonymous with weights in the case where 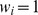): 
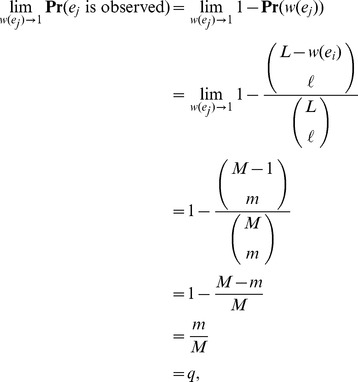
where *q* is the proportion of sampled links. Thus, when the weights on edges tends to 1, our Horvitz-Thompson estimator is 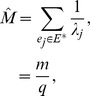
which recovers our previous result for scaling of edges when sampling by links. The scaling of network statistics is demonstrated in Fig. S13. The results of applying our estimation techniques to the node strength and degree distribution are shown in Figs. S14 and S15. The relative error incurred for the predicted number of edges is presented in Tables S18 and S19 in Materials S1.

Having found suitable predictors for *N* and *M*, the average degree may be predicted by, 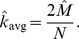



Applying these scaling techniques, we obtain reasonably low error for both networks in both experiments 1 and 2 (Tables S20 and S21 in Materials S1).

To estimate *k*_max_, we recognize that the observed max degree will need to be scaled by roughly the proportion of missing edges. Using 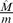 as our scaling factor, we find relatively high error for both networks (Tables S22 and S23). This is due to errors in the 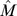 hindering accuracy in 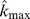.

### Estimating the size of the Twitter interactome

We now consider the weighted, directed network of replies whereby a link from node 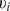 to node 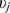 represents the existence of at least one reply directed from 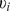 to 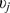 and the weight on this edge represents the number of messages sent in the time period under consideration. We apply our methods to reply networks constructed from tweets gathered during the ten week period from September 9, 2008 to November 17, 2008, a period for which we have a substantially higher percentage of all authored messages.

For each of these weeks, we receive between 20–55% of all messages posted on Twitter and similarly believe that we receive approximately 20–55% of all replies posted in this period (Table S24 in Materials S1). We apply our previously developed methods to estimate the number of nodes, edges, strengths on these edges, average degree, max degree, and distribution of node strength. To help validate our predictions, we also predict the number of nodes, edges, average degree, and max degree by performing 100 sampling experiments in which a proportion *q* of the observed messages used for subnetwork construction. These sampling experiments essentially “hide” some of the messages from our view and thus allow us to consider how further subsampling impacts the inferred networks statistics. Curve fitting over this region of *q* allows us to extrapolate the network statistic to a predicted value over increased percentages of observed messages. We use this to validate with our estimated statistic using the methods from the previous section.

#### Number of nodes

**Figure 5:**
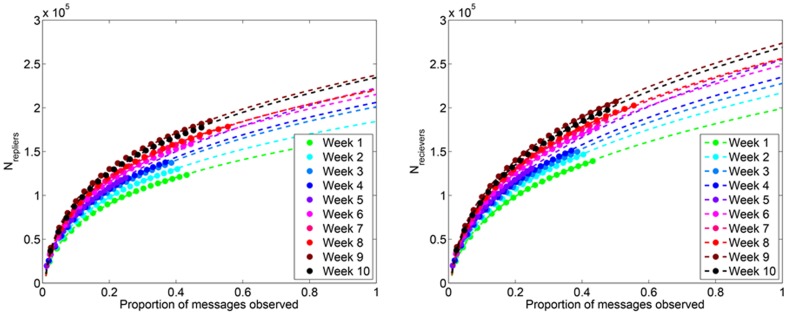
Number of nodes in Twitter reply subnetworks. (Left) The quantity *N*_repliers_ is shown for Weeks 1 to 10, where each data point (dot) represents the average over 100 simulated subsampling experiments. The dashed line represents the best fitting model of the form *N*_repliers_ = *ax^b^* to the observed data. We extrapolate this model to predict *N*_repliers_. (Right) The same as panel, except for *N*_receivers._

**Figure 6:**
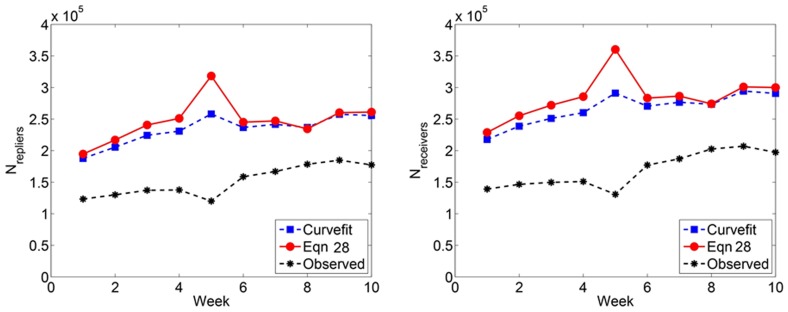
Predicted number of nodes in Twitter reply networks. The number of nodes observed for each week is depicted, along with the predicted number of nodes obtained from curve fitting (Fig. 5) and Equation 28. The predicted number of nodes is nearly double the number of observed nodes. The relatively low proportion of messages received for Week 5 (<25%) may be creating greater inaccuracies in the predictors for that week.

Since our reply networks are directed, we consider both the number of nodes which make a reply (*N*_repliers_) and the number of nodes which receive a reply (*N*_receiver_). As expected from our previous discussion, the number of nodes scales nonlinearly with the proportion of observed messages (Fig. 5). We fit models of the form N = *ax^b^* to observed data and in doing so find an excellent fit (*R* ^2^≈0.99) for all weeks over the subsampled region (Fig. 5). Extrapolating these fitted models to *q* = 1, we find excellent agreement with our predicted number of nodes obtained from Equations 29 and 30. The predicted number of nodes from both methods agree to within ±5%. Figure 6 reveals that the predicted number of nodes is nearly double the number of observed nodes.

#### Strength of nodes

**Figure 7:**
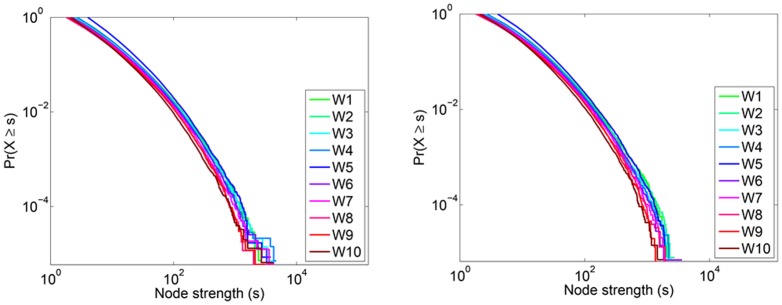
Predicted *P_s_* for Twitter reply networks. (Left) The node strength distribution for in-coming interactions. (Right) The node strength distribution out-going interactions. In both cases, the distribution is heavy tailed, but falls off faster than would be expected in a scale-free distribution.

**Figure 8:**
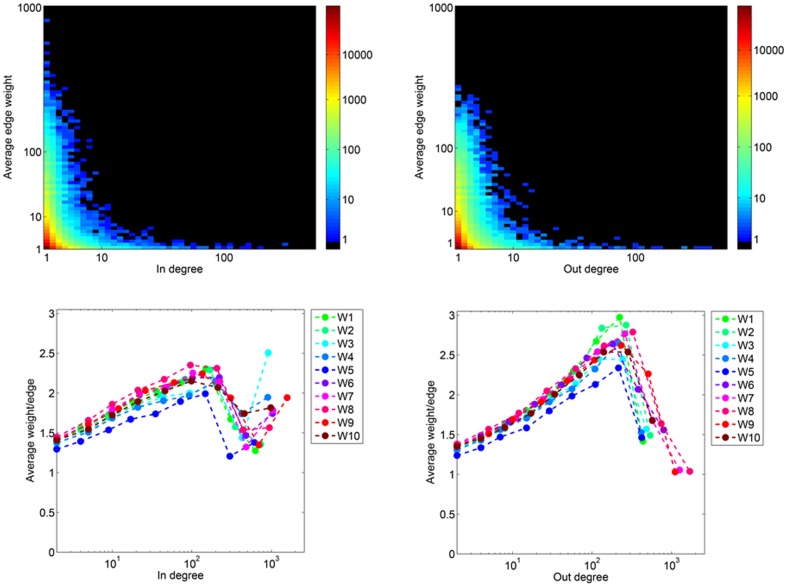
In, Out-degree vs. Average edge weight for Twitter reply networks. (Top, left) The average in-coming edge weight for each node of degree *k* is depicted in a logarithmically binned heatmap. (Top, right) The same as (a), except for out-going edges. (c.) The average weight per edge for in-coming edges as a function of *k*_in_ shows a gradual increase to *k*_in_≈10^2^ with a peak of approximately 2.2 interactions per edge. (d.) The average weight per edge for out-going edges as a function of *k*_out_ shows a gradual increase to *k*_out_≈10^2^ with a peak of between 2.5 and 3 interactions per edge.

Figure 7 depicts a log-log plot of the predicted node strength distribution. This plot reveals that there are fewer nodes in the high strength region than would be expected in a scale-free distribution. Figure 8 reveals that low degree nodes dominate the dataset and that many of these low degree nodes often have low average edge weight (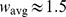). We find a peak in the average weight per edge as a function of degree around 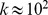. This peak is more pronounced for out-going edges. Beyond this value, a limiting factor may prevent increases in the weight per edge, a result also noted by Gonçalves et al. [48].

#### Number of edges

**Figure 9:**
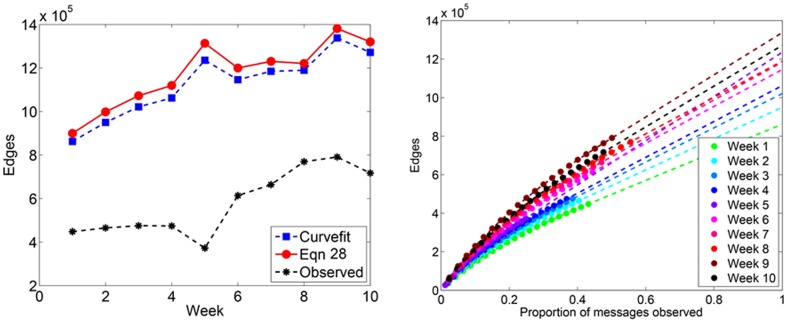
Predicted number of edges in Twitter reply networks. (Left) A small proportion of observed messages for Week 5 (<25%) may explain the spike in the estimated number of edges for that week. (Right) Each data point represents the number of directed edges observed, averaged over 100 simulated subsampling experiments. The dashed line extrapolates the predicted number of edges for greater proportions of sampled data.

**Figure 10:**
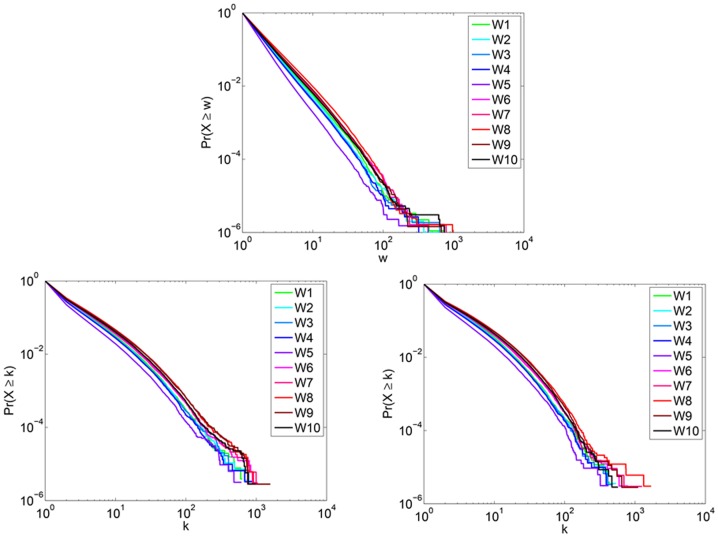
Predicted edge weight and degree distributions for Twitter reply networks. (Top) The predicted edge weight distribution. (Bottom, left) Predicted *Pr*(*k*_in_) and (Bottom, right) *Pr*(*k*_out_) for Twitter reply networks.

The number of edges can be predicted using Equations 31 and 32. We present our results in Figure 9. In all cases, the number of edges increases throughout the period of the study. Figure 10 depicts the predicted edge weight and degree distributions. The edge weight distribution shows that very few (<.001%) edges have weight greater than 10^2^. The degree distribution of the observed subnetwork can be rescaled by reassigning nodes of degree *k*, to nodes of degree 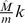. Figure 10 demonstrates a slightly heavier tail in the in-degree distribution as compared to the out-degree distribution. The degree distribution reveals that fewer than .01% of the nodes have more than 10^2^ distinct neighbors. This value is approximately Dunbar's number, a value suggested to be the upper limit on the number of active social contacts for humans [49].

#### Average degree

**Figure 11:**
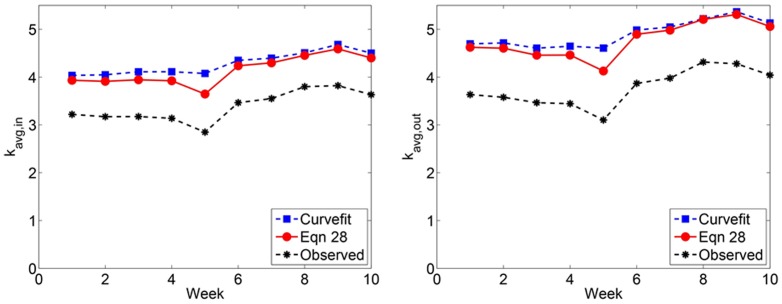
(Left) Predicted *k_avg,in_* and (Right) *k_avg,out_* in Twitter reply networks.

**Figure 12:**
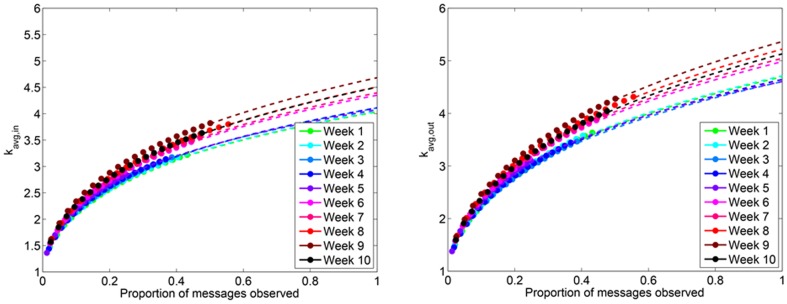
(Left) *k*_avg,in_ and (Right) *k*_avg,in_ for Twitter reply networks. Each data point represents the observed average in- and out-degree, averaged over 100 simulated subsampling experiments. The dashed line extrapolates the predicted number of edges for greater proportions of sampled data.

Once the number of nodes and edges have been predicted for the network, we may simply compute the average degree as 
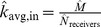
 and 
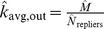
. Upon doing so, we find that the average degree for Twitter reply networks is between 4 and 5 (Fig. 11). We find that the average in-degree is less than the average out-degree (Fig. 12).

#### Maximum degree

**Figure 13:**
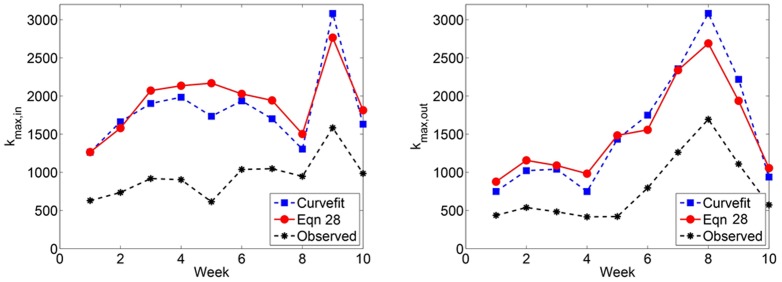
(Left) Predicted *k*_max,in_ and (Right) *k*_max,out_ in Twitter reply networks.

**Figure 14:**
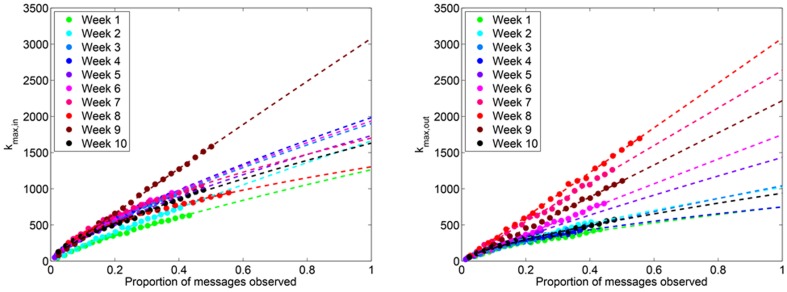
(Left) *k*_max,in_ and (Right) *k*_max,in_ for Twitter reply networks. Each data point represents the observed maximum in- and out-degree, averaged over 100 simulated subsampling experiments. The dashed line extrapolates the predicted number of edges for greater proportions of sampled data.

The maximum degree simply scales in proportion to the probability of edge inclusion. Since the probability of edge inclusion is no longer *q*, as in the case of sampling by links, we may approximate the probability of edge inclusion by 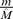 and thus 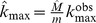. The predicted maximum degree for Twitter reply networks is shown in Figures 13 and 14.

## Discussion

Network measures derived from empirical observations will often be poor estimators of the true underlying network structure of the system. We have explored four sampling regimes: (1) subnetworks induced on randomly sampled nodes, (2) subnetworks obtained when all nodes are known and some links fail or are hidden, (3) subnetworks generated from randomly sampled links, and (4) weighted subnetworks generated by randomly sampled interactions. We have described how network statistics scale under these regimes via sampling experiments on simulated and empirical networks. Our paper advances an understanding of how network statistics scale, and more importantly how to correct for missing data when the proportion of missing nodes, links or interactions is known.

A major obstacle to generating scaling techniques for subnetworks generated by sampled links or interactions has previously been the lack of a practical method for estimating the true degree distribution or node strength distribution. Problematically, the random selection of links creates a biased sample of nodes whereby hubs are more likely to be detected, and nodes of small degree are more likely to go undetected. Although scaling methods have been suggested, they are based on knowledge of (or a reasonable estimate of) the degree or node strength distribution [3]. In this paper, we have overcome this obstacle by our proposed scaling techniques for the degree distribution and apply this to several simulated and empirically derived networks with reasonably good results.

Very few studies have addressed the missing data problem in empirically studied networks, such as those constructed from tweets. An exception is work by Morstatter et al. [2] who compared network statistics for the current Twitter's Spritzer (≈1% of all tweets) to the full Firehose (100% of all tweets), however no methods for scaling from data collected via the API were suggested.

We concluded our work by applying our derived scaling methods to Twitter reply networks. Our work supports Dunbar's hypothesis which suggests that individuals maintain an upper limit of roughly 100–150 contacts each week [49]. Further evidence for this hypothesis comes from previous work in link prediction. Bliss et al. [50] detect the Resource Allocation index to often evolve a large, positive weight–thus contributing heavily (and positively) in the prediction of new links. This index considers the amount of time and attention one individual has as a “social resource” to spend in the social network and assumes that each node will distribute its resource equally among all neighbors. Although the presence of hubs is suggestive of preferential attachment, it is clear that the constraints of time and attention limit truly scale-free behavior in weekly Twitter reply networks. We find that the number of individuals who make replies is less than the number of individuals who receive replies.

One limitation of our work is that our scaling methods are based upon the assumption that the sampling fraction, *q* is known, while in practice this need not be the case. In cases where one may establish an upper and lower bound for *q*, our methods could be used to help establish bounds for the predicted network measures. In some cases, particularly when sampling by links or interactions, small changes in *q* may have relatively little impact on the predicted statistics, especially for large *q*. Future work that seeks to classify subnetworks by network class based on signature subsampling properties may also prove to be fruitful. With some knowledge of network class or generative model, methods for estimating *q* may be possible. Additionally, efforts to predict structural holes in networks from localized information may also greatly advance the field [51].

To our knowledge, this is the first attempt provide scaling methods for *k*_max_. While our scaling techniques for predicting *k*_max_ perform well for several networks, they did not perform as well on simulated networks with a regularized structure. Our rewiring probability for the simulated Small world networks was quite low, with *p* = 0.1. Our methods perform well on other networks which are known to exhibit to Small world structure, such as our empirical networks Powergrid and *C. elegans*. Future work which detects and accounts for motif distributions may improve upon our efforts here.

With an increased interest in large, networked datasets, we hope that continued efforts will aid in the understanding of how subsampled network data can be used to infer properties of the true underlying system. Our methods advance the field in this direction, not only adding to the body of literature surrounding sampling issues and Twitter's API [2], but also to the growing body of literature on incomplete network data.

## Supporting Information

Materials S1
**Supporting figures and tables.** Derivation of Equation (2). Figure S1: Scaling of statistics for simulated subnetworks induced on sampled nodes. Figure S2: Scaling of statistics for empirical subnetworks induced on sampled nodes. Figure S3: CCDF distortion for subnetworks induced on sampled nodes. Figure S4: Predicted CCDF from subnetworks induced on sampled nodes. Figure S5: Scaling of subnetwork statistics for simulated networks obtained by failing links. Figure S6: Scaling of subnetwork statistics for empirical networks obtained by failing. Figure S7: CCDF distortion for subnetworks obtained by failing links. Figure S8: Predicted CCDF from subnetworks obtained by failing links. Figure S9: Scaling of subnetwork statistics for simulated networks induced on sampled links. Figure S10: Scaling of subnetwork statistics for empirical networks induced on sampled links. Figure S11: CCDF distortion for subnetworks induced on sampled links. Figure S12: Predicted CCDF from subnetworks induced on sampled links. Figure S13: Scaling of subnetwork statistics for simulated networks induced on sampled interactions. Figure S14: Predicted node strength distribution for weighted, simulated networks. Figure S15: Predicted degree distribution for weighted, simulated networks. Figure S16: Kolmogorov-Smirnov two sample test for true CDF and predicted CDF from subnetworks induced on sampled nodes. Figure S17: Kolmogorov-Smirnov two sample test for true CDF and predicted CDF from subnetworks obtained by failing links. Figure S18: Kolmogorov-Smirnov two sample test for true CDF and predicted CDF from subnetworks generated by sampled links. Table S1: Error in 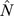 when sampling by nodes. Table S2: Error in 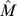 when sampling by nodes. Table S3: Error in 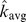 when sampling by nodes. Table S4: Error in 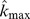 when sampling by nodes. Table S5: Error in 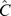 when sampling by nodes. Table S6: Error in 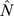 when failing links. Table S7: Error in 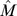 when failing links. Table S8: Error in 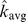 when failing links. Table S9: Error in 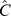 when failing links. Table S10: Error in 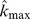 when failing links. Table S11: Error in 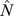 when sampling by links. Table S12: Error in 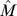 when sampling by links. Table S13: Error in 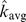 when sampling by links. Table S14: Error in 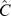 when sampling by links. Table S15: Error in 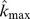 when sampling by links. Table S16: Error in 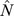 when sampling by interactions in an Erdös-Rényi random graph. Table S17: Error in 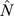 when sampling interactions in a Scale-free weighted network. Table S18: Error in 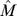 when sampling by interactions in an Erdös-Rényi random graph. Table S19: Error in 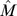 when sampling interactions in a Scale-free weighted network. Table S20: Error in 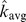 when sampling by interactions in an Erdös-Rényi random graph. Table S21: Error in 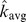 when sampling interactions in a Scale-free weighted network. Table S22: Error in 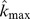 when sampling by interactions in an Erdös-Rényi random graph. Table S23: Error in 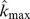 when sampling interactions in a Scale-free weighted network. Table S24: Number of messages from September 2008-November 2009.(PDF)
