# Supplementary material for: Estimation of Global Network Statistics from Incomplete Data
Source: PLoS One. 2014 Oct 22;9(10):e108471. doi: 10.1371/journal.pone.0108471 (PMC4206292; doi:10.1371/journal.pone.0108471)
Supplement: Materials S1 — Supporting figures and tables. Derivation of Equation (2). Figure S1: Scaling of statistics for simulated subnetworks induced on sampled nodes. Figure S2: Scaling of statistics for empirical subnetworks induced on sampled nodes. Figure S3: CCDF distortion for subnetworks induced on sampled nodes. Figure S4: Predicted CCDF from subnetworks induced on sampled nodes. Figure S5: Scaling of subnetwork statistics for simulated networks obtained by failing links. Figure S6: Scaling of subnetwork statistics for empirical networks obtained by failing. Figure S7: CCDF distortion for subnetworks obtained by failing links. Figure S8: Predicted CCDF from subnetworks obtained by failing links. Figure S9: Scaling of subnetwork statistics for simulated networks induced on sampled links. Figure S10: Scaling of subnetwork statistics for empirical networks induced on sampled links. Figure S11: CCDF distortion for subnetworks induced on sampled links. Figure S12: Predicted CCDF from subnetworks induced on sampled links. Figure S13: Scaling of subnetwork statistics for simulated networks induced on sampled interactions. Figure S14: Predicted node strength distribution for weighted, simulated networks. Figure S15: Predicted degree distribution for weighted, simulated networks. Figure S16: Kolmogorov-Smirnov two sample test for true CDF and predicted CDF from subnetworks induced on sampled nodes. Figure S17: Kolmogorov-Smirnov two sample test for true CDF and predicted CDF from subnetworks obtained by failing links. Figure S18: Kolmogorov-Smirnov two sample test for true CDF and predicted CDF from subnetworks generated by sampled links. Table S1: Error in when sampling by nodes. Table S2: Error in when sampling by nodes. Table S3: Error in when sampling by nodes. Table S4: Error in when sampling by nodes. Table S5: Error in when sampling by nodes. Table S6: Error in when failing links. Table S7: Error in when failing links. Table S8: Error in when failing links. Table S9: Error in when fa [file pone.0108471.s001.pdf]

S1 Supporting figures and tables

Derivation of Equation (2) Restating Equation (1) for convenience, with $K = k_{\max}$ for ease of notation, we have

$$\tilde{P}_k = \sum_{i=k}^{K} \binom{i}{k} q^k \, (1-q)^{i-k} \, P_i.$$

Assume a finite network with maximum degree, K. Equation (1) may be rewritten as:

$$\begin{bmatrix} \tilde{P}_1 \\ \tilde{P}_2 \\ \tilde{P}_3 \\ \vdots \\ \tilde{P}_{K-2} \\ \tilde{P}_{K-1} \\ \tilde{P}_K \end{bmatrix} = \begin{bmatrix} \binom{1}{1}q^1\,(1-q)^{1-1} + & \binom{2}{1}q^1\,(1-q)^{2-1} + & \binom{3}{1}q^1\,(1-q)^{3-1} & + & \dots & + & \binom{K-1}{1}q^1\,(1-q)^{K-2} + & \binom{K}{1}q^1\,(1-q)^{K-1} \\ & \binom{2}{2}q^2\,(1-q)^{2-2} + & \binom{3}{2}q^2\,(1-q)^{3-2} + & + & \dots & + & \binom{K-1}{2}q^2\,(1-q)^{K-3} + & \binom{K}{2}q^2\,(1-q)^{K-2} \\ & & \binom{3}{3}q^3\,(1-q)^{3-3} & + & \dots & + & \binom{K-1}{3}q^3\,(1-q)^{K-4} + & \binom{K}{3}q^3\,(1-q)^{K-3} \\ & & & & & & & \vdots \\ & & & & \ddots & & \vdots & \vdots \\ & & & & & & \binom{K-1}{K-1}q^{K-1}\,(1-q)^0 + & \binom{K}{K-1}q^{K-1}\,(1-q)^1 \\ & & & & & & & \binom{K}{K}q^K\,(1-q)^0 \end{bmatrix} \begin{bmatrix} P_1 \\ P_2 \\ P_3 \\ \vdots \\ P_{K-2} \\ P_{K-1} \\ P_K \end{bmatrix}$$

Back solving for P_K yields:

$$P_k = \frac{1}{q^K} \tilde{P}_K$$

Continuing, we solve for P_{K-1}.

$$\begin{aligned} \tilde{P}_{K-1} &= \binom{K-1}{K-1} q^{K-1}\,(1-q)^0\, P_{K-1} + \binom{K}{K-1} q^{K-1}\,(1-q)^1\, P_K \\ P_{K-1} &= \frac{\tilde{P}_{K-1} - \binom{K}{K-1} q^{K-1}(1-q) P_K}{q^{K-1}} \\ P_{K-1} &= \frac{\tilde{P} + K - 1 - \binom{K}{K-1} q^{K-1}(1-q)\left(\frac{\tilde{P}_K}{q^K}\right)}{q^{K-1}} \\ P_{K-1} &= \frac{\tilde{P}_{K-1}}{q^{K-1}} - \frac{K(1-q)}{q^K} \tilde{P}_K \end{aligned}$$

Solving for P_{K-2} yields,

$$\begin{aligned} \tilde{P}_{K-2} &= \binom{K-2}{K-2} q^{K-2}\,(1-q)^0\, P_{K-2} + \binom{K-1}{K-2} q^{K-2}\,(1-q)^1\, P_{K-1} + \binom{K}{K-2} q^{K-2}\,(1-q)^2\, P_K \\ P_{K-2} &= \frac{\tilde{P}_{K-2} - \binom{K-1}{K-2} q^{K-2}\,(1-q)^1\, P_{K-1} - \binom{K}{K-2} q^{K-2}\,(1-q)^2\, P_K}{q^{K-2}} \\ P_{K-2} &= \frac{\tilde{P}_{K-2}}{q^{K-2}} - (K-1)(1-q)P_{K-1} - \frac{K(K-1)}{2}(1-q)^2 P_K \end{aligned}$$

Now, using our previous results, we have

$$P_{K-2} = \frac{\tilde{P}_{K-2}}{q^{K-2}} - (K-1)(1-q)\left(\frac{\tilde{P}_{K-1}}{q^{K-1}} - \frac{K(1-q)}{q^K}\tilde{P}_K\right) - \frac{K(K-1)}{2}(1-q)^2\left(\frac{\tilde{P}_K}{q^K}\right) \tag{1}$$

$$P_{K-2} = \frac{\tilde{P}_{K-2}}{q^{K-2}} - \frac{(K-1)(1-q)}{q^{K-1}}\tilde{P}_{K-1} + \frac{K(K-1)(1-q)^2}{q^K}\tilde{P}_K - \frac{K(K-1)(1-q)^2}{2q^K}\tilde{P}_K \tag{2}$$

$$P_{K-2} = \frac{\tilde{P}_{K-2}}{q^{K-2}} - \frac{(K-1)(1-q)}{q^{K-1}}\tilde{P}_{K-1} + \frac{K(K-1)(1-q)^2}{2q^K}\tilde{P}_K \tag{3}$$

Proceeding, we may generalize our result as

$$\hat{P}_{K-n} = \sum_{i=K-n}^{K} \frac{(-1)^{i-(K-n)}\binom{i}{K-n}(1-q)^{i-(K-n)}}{q^i}\tilde{P}_i, \tag{4}$$

which is

$$\hat{P}_k = \sum_{i=k}^{K} \frac{(-1)^{i-k}\binom{i}{k}(1-q)^{i-k}}{q^i}\tilde{P}_i, \tag{5}$$

with $k - K - n$.

Verification of Equation 2. We will demonstrate that our derivation for the predicted degree distribution, $\hat{P}_k$, agrees with the true degree distribution P_k. Observe that

$$\begin{aligned}
\hat{P}_k &= \sum_{i=k}^{K} \frac{(-1)^{i-k}\binom{i}{k}(1-q)^{i-k}}{q^i} \tilde{P}_i, \\
&= \underbrace{\tilde{P}_k}_{i=k} - \underbrace{\frac{\binom{k+1}{k}(1-q)^1}{q^{k+1}} \tilde{P}_{k+1}}_{i=k+1} + \ldots \underbrace{\frac{(-1)^{K-k}\binom{K}{k}(1-q)^{K-k}}{q^K} \tilde{P}_K}_{i=K} \\
&= \underbrace{\sum_{j=k}^{K} \binom{j}{k} q^k (1-q)^{j-k} P_j}_{i=k} - \underbrace{\frac{\binom{k+1}{k}(1-q)^1}{q^{k+1}} \sum_{j=k+1}^{K} \binom{j}{k+1} q^{k+1} (1-q)^{j-(k+1)} P_j}_{i=k+1} \\
&\quad + \ldots \underbrace{\frac{(-1)^{K-k}\binom{K}{k}(1-q)^{K-k}}{q^K} \sum_{j=K}^{K} \binom{j}{K} q^K (1-q)^{j-K} P_j}_{i=K} \\
&= \underbrace{\sum_{j=k}^{K} \binom{j}{k} q^k (1-q)^{j-k} P_j}_{i=k} - \underbrace{\binom{k+1}{k}(1-q)^1 \sum_{j=k+1}^{K} \binom{j}{k+1} (1-q)^{j-(k+1)} P_j}_{i=k+1} \\
&\quad + \ldots \underbrace{(-1)^{K-k}\binom{K}{k}(1-q)^{K-k} \sum_{j=K}^{K} \binom{j}{K} (1-q)^{j-K} P_j}_{i=K}
\end{aligned}$$

Expanding the sums in all terms and collecting powers of $(1-q)$ yields

$$\begin{aligned}
\hat{P}_k = P_k &+ \binom{k+1}{k}(1-q)P_{k+1} + \binom{k+2}{k}(1-q)^2 P_{k+2} + \ldots + \binom{K}{k}(1-q)^{K-k} P_K \\
&- \binom{k+1}{k}(1-q)P_{k+1} - \binom{k+1}{k}\binom{k+2}{k+1}(1-q)^2 P_{k+2} - \ldots - \binom{k+1}{k}\binom{K}{k}(1-q)^{K-k} P_K \\
&\qquad \vdots \\
&\qquad (-1)^{K-k}\binom{K}{k}(1-q)^{K-k} P_K
\end{aligned}$$

For each power of $(1-q)$, we have

$$\begin{aligned}
\sum_{i=0}^{a} (-1)^a \binom{k+i}{k}\binom{k+b}{k+i}(1-q)^a P_{k+a} &= \sum_{i=0}^{a} (-1)^a \binom{k+a}{k}\binom{a}{i}(1-q)^a P_{k+a} \\
&= \binom{k+a}{k}\left(\sum_{i=0}^{a} (-1)^a \binom{a}{i}\right)(1-q)^a P_{k+a} \\
&= \begin{cases} \binom{k+a}{k}(0)(1-q)^a P_{k+a}, & \text{if } a \geq 1 \\ \binom{k+a}{k}(1)(1-q)^a P_{k+a}, & \text{if } a = 0 \end{cases} \\
&= \begin{cases} 0, & \text{if } a \geq 1 \\ P_k, & \text{if } a = 0 \end{cases}
\end{aligned}$$

whereby we have made use of the binomial theorem $(x+y)^a = \sum_{i=0}^{a} \binom{a}{i}(x^{a-i}y^i$ for $x = 1, y = -1$ which implies that $0 = \sum_{i=0}^{a}(-1)^a\binom{a}{i}$, for $a \geq 1$. Thus, all terms of $\hat{P}_k$ cancel, except for the P_k and so $\hat{P}_k = P_k$. □

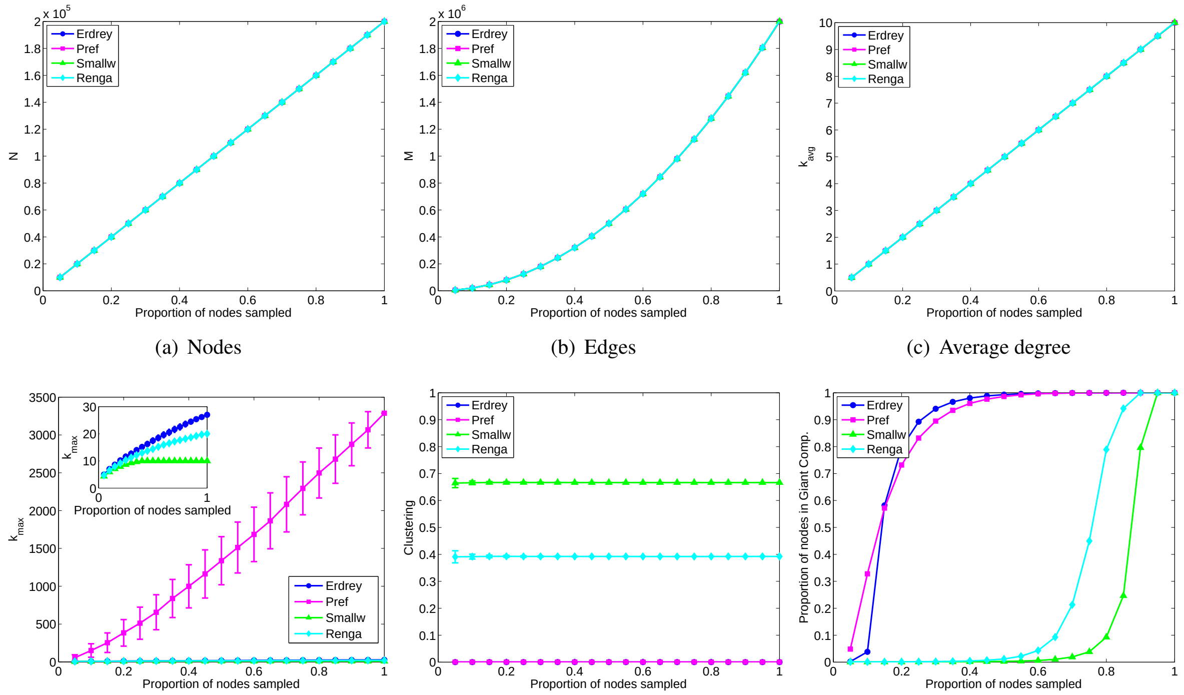

(a) Nodes (b) Edges (c) Average degree

(d) Max degree (e) Clustering (f) Prop. of nodes in Giant Component

Figure S1: Scaling of statistics for simulated subnetworks induced on sampled nodes. (a.) The number of nodes in a subnetwork sampled by nodes scales as $n = qN$ precisely because only qN nodes are selecting during subsampling. (b.) The number of edges scales as $m \approx M\frac{n(n-1)}{N(N-1)} \approx Mq^2$, for $n \gg 1$ and $N \gg 1$. (c.) The average degree scales linearly with the proportion of nodes subsampled. (d.) The scaling of the max degree is dependent on network type. For networks with few large hubs, $k^{\text{obs}}_{k_{\text{max}}} \approx qk_{\text{max}}$. For networks exhibiting a nontrivial number of nodes with degrees relatively close to k_{max}, the max. degree scales nonlinearly. (e.) The clustering coefficient [21] shows little variation with respect to q as suggested by the analytical result from Frank [43]. This suggests that $\hat{C} \approx C_{\text{obs}}$. (f.) The proportion of nodes in the giant component increases with the proportion of nodes sampled. For the random graphs (Erdrey and Pref) there is a critical point corresponding to the approximate sampling level corresponding to when $k^{\text{obs}}_{\text{avg}} > 1$. The thresholds for Small World and Range dependent networks are much higher due to the uniformity of the motif distribution in these networks. Markers indicates the mean over 100 simulations. Error bars showing one standard deviation are too small to see, except for (d.).

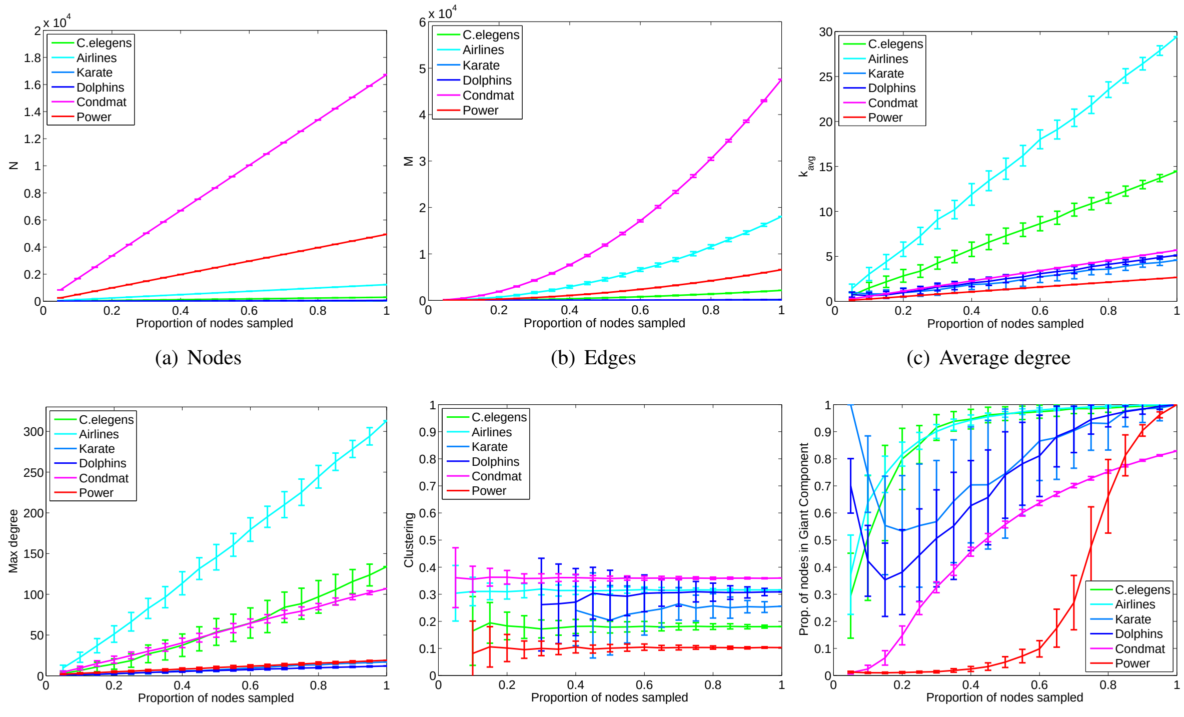

(a) Nodes (b) Edges (c) Average degree

(d) Max degree (e) Clustering (f) Prop. of nodes in Giant Component

Figure S2: Scaling of statistics for empirical subnetworks induced on sampled nodes. (a.) The number of nodes scales as $n = qN$ precisely because only qN nodes are selecting during subsampling. (b.) The number of edges scales as $m \approx M\frac{n(n-1)}{N(N-1)} \approx Mq^2$, where q is the proportion of nodes subsampled. (c.) The average degree scales as $k_{\text{avg}}^{\text{obs}} \approx qk_{\text{avg}}^{\text{true}}$. (d.) The max degree scales roughly linearly as $k_{\text{max}}^{\text{obs}} \approx qk_{\text{max}}^{\text{true}}$. (e.) The clustering coefficient [21] shows little variation with respect to q as suggested by the analytical result from Frank [43], $\hat{C} \approx C_{\text{obs}}$. (f.) Large networks, such as the Powergrid and Condensed Matter author collaboration networks show the expected transition to the giant component as q increases corresponding to when $k_{\text{avg}}^{\text{obs}} > 1$. Smaller networks, such as the Karate club and Dolphin network show a high proportion of nodes in the giant component, for low q because the subnetwork generated for these levels of q contains fewer than 10 nodes (i.e., the network is degenerate).

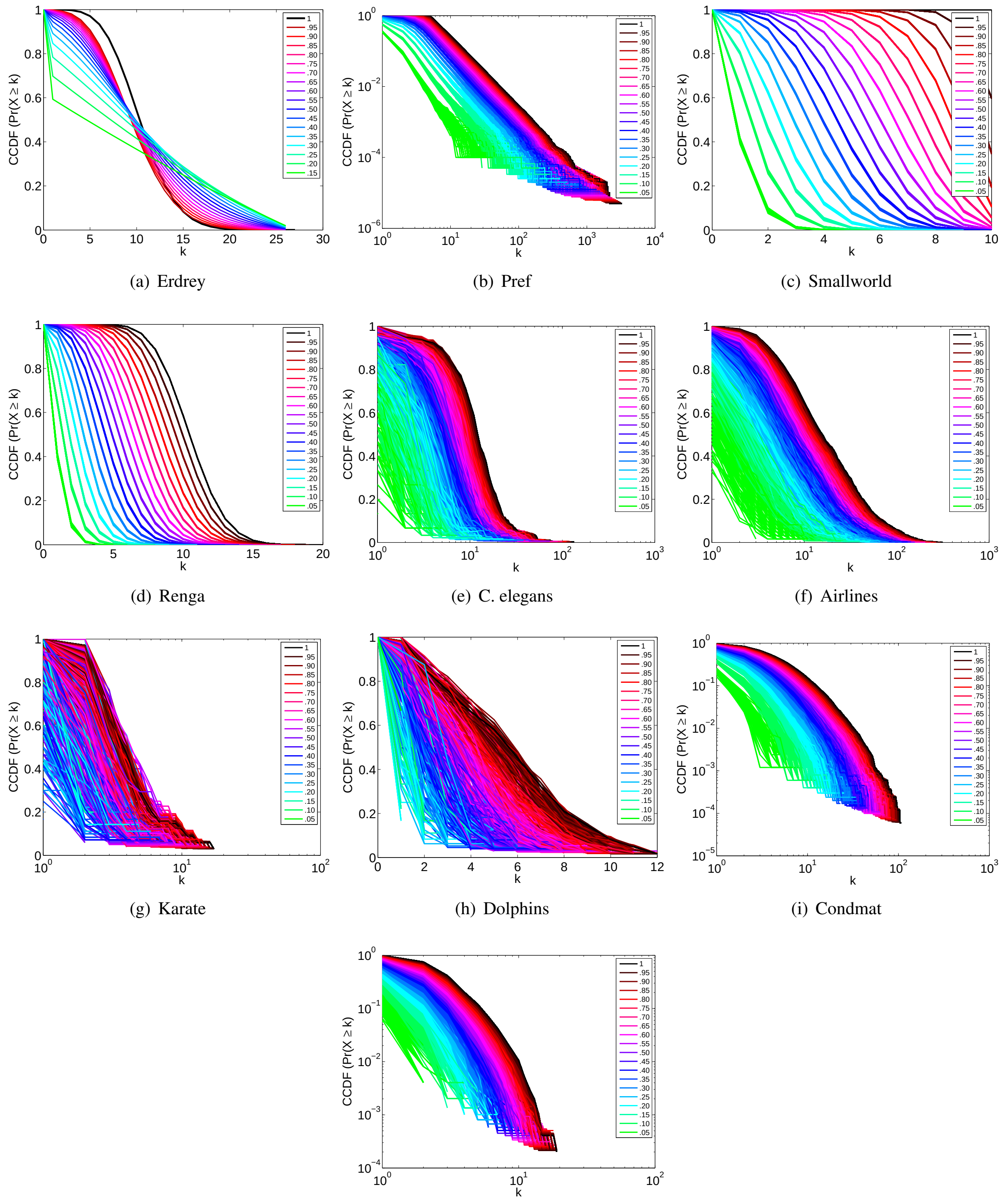

(a) Erdrey (b) Pref (c) Smallworld (d) Renga (e) C. elegans (f) Airlines (g) Karate (h) Dolphins (i) Condmat (j) Powergrid

Figure S3: CCDF distortion for subnetworks induced on sampled nodes. Subnetwork degree distributions do not capture the true degree distribution, especially for small q.

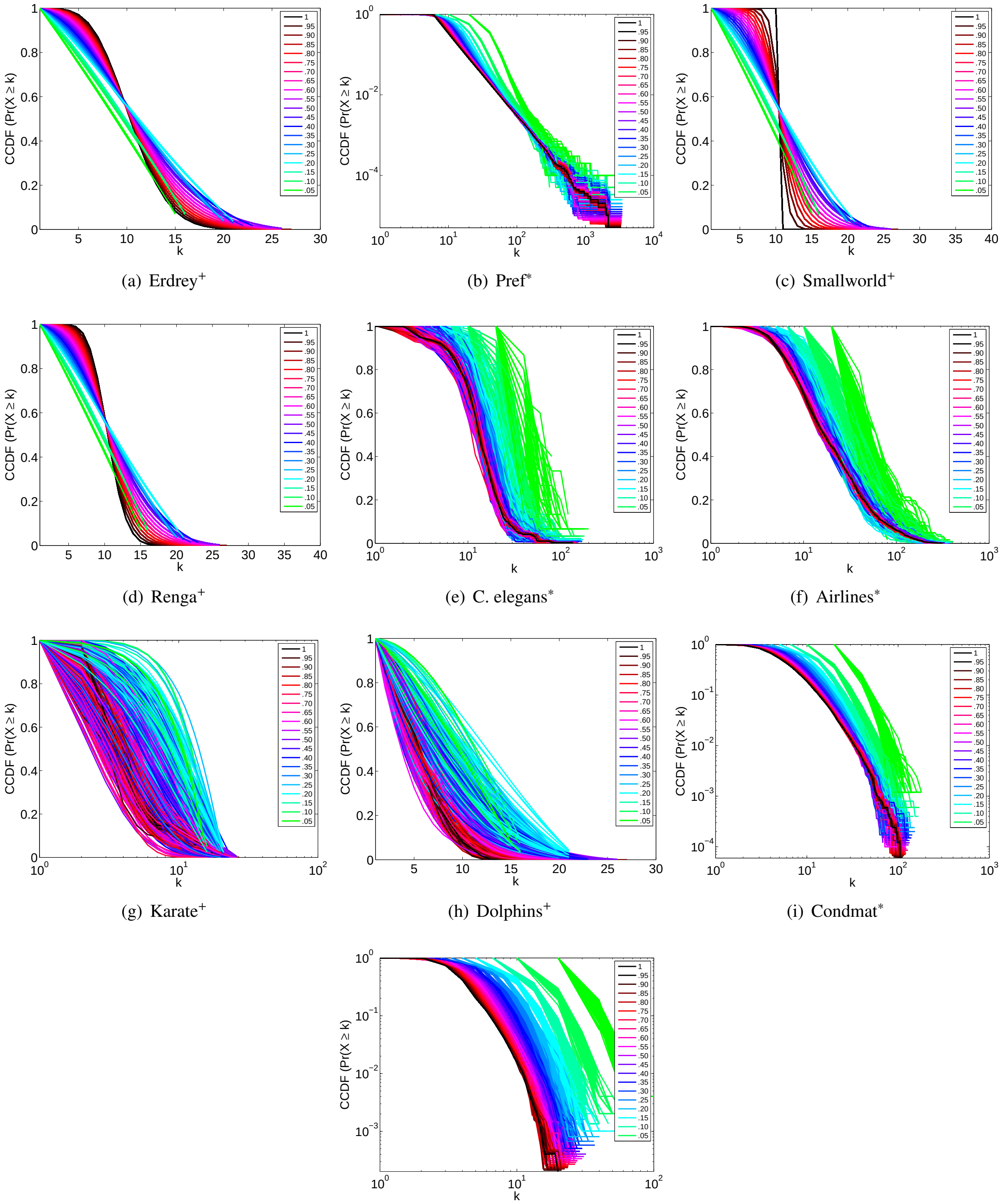

(a) Erdrey+

(b) Pref*

(c) Smallworld+

(d) Renga+

(e) C. elegans*

(f) Airlines*

(g) Karate+

(h) Dolphins+

(i) Condmat*

(j) Powergrid*

Figure S4: Predicted CCDF from subnetworks induced on sampled nodes. The predicted CCDF shows relatively good agreement with the true CCDF for most networks. Karate club and Dolphins exhibit significant deviation, possible due to the small number of nodes in these networks. Networks designated with + utilized Equation [16] and those designated with with * utilized Equation [18].

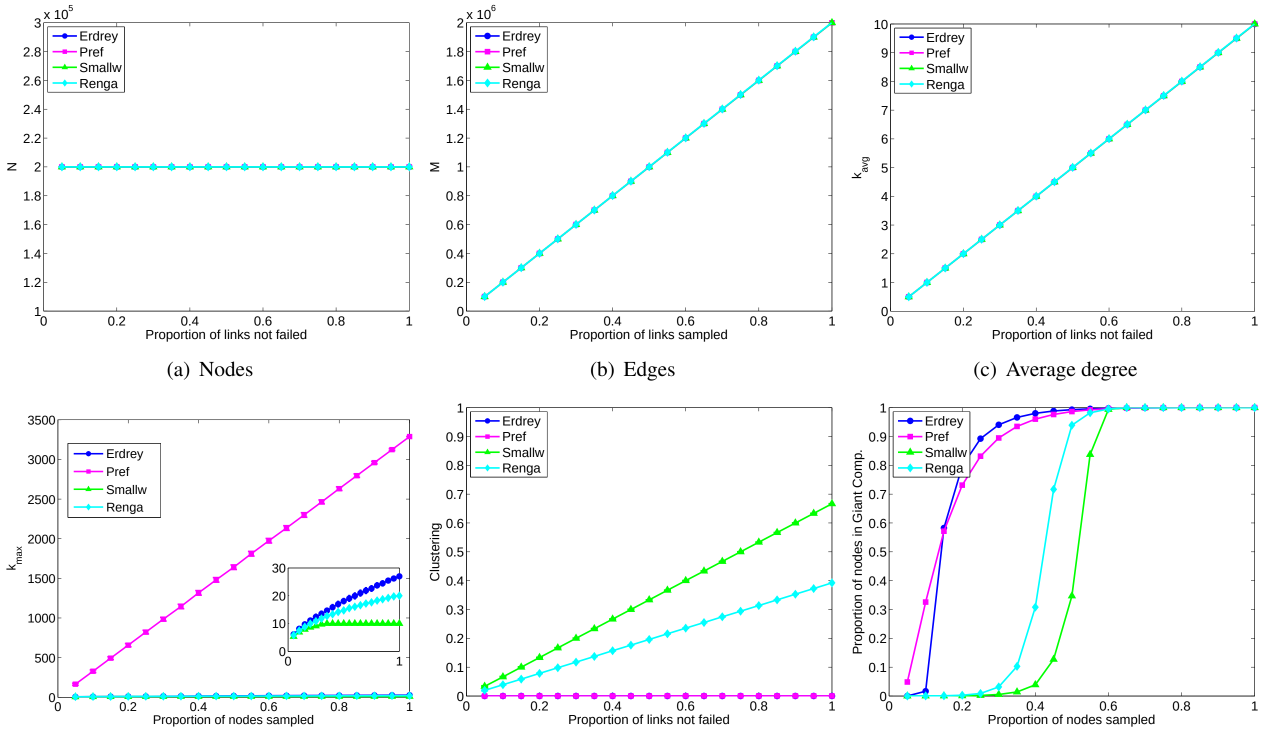

(a) Nodes (b) Edges (c) Average degree

(d) Max degree (e) Clustering (f) Prop. of nodes in Giant Component

Figure S5: Scaling of subnetwork statistics for simulated networks obtained by failing links. (a.) When all nodes are known q links are observed through sampling, the sample statistic for the number of nodes n equals the true number of nodes N. It should be noted, though, that some nodes of degree 0 may be observed and these are counted as nodes (not discarded). (b.) The number of edges scales linearly as $M_{\mathrm{obs}} = qM$. (c.) The average degree scales linearly as $k_{\mathrm{avg}}^{\mathrm{obs}} = \frac{k_{\mathrm{avg}}^{\mathrm{true}}}{q}$. (d.) The max degree scales linearly for Pref, but nonlinearly for other networks which have several nodes with degree similar to k_{max}. (e.) Clustering scales roughly linearly with q. (f.) The percolation threshold for random graphs (Erdös-Rényi and Preferential attachment) roughly corresponds to the q for which $k_{\mathrm{avg}} \geq 1$. Smallworld and Renga show more fragility and have a threshold which is closer to $q \approx 0.4$.

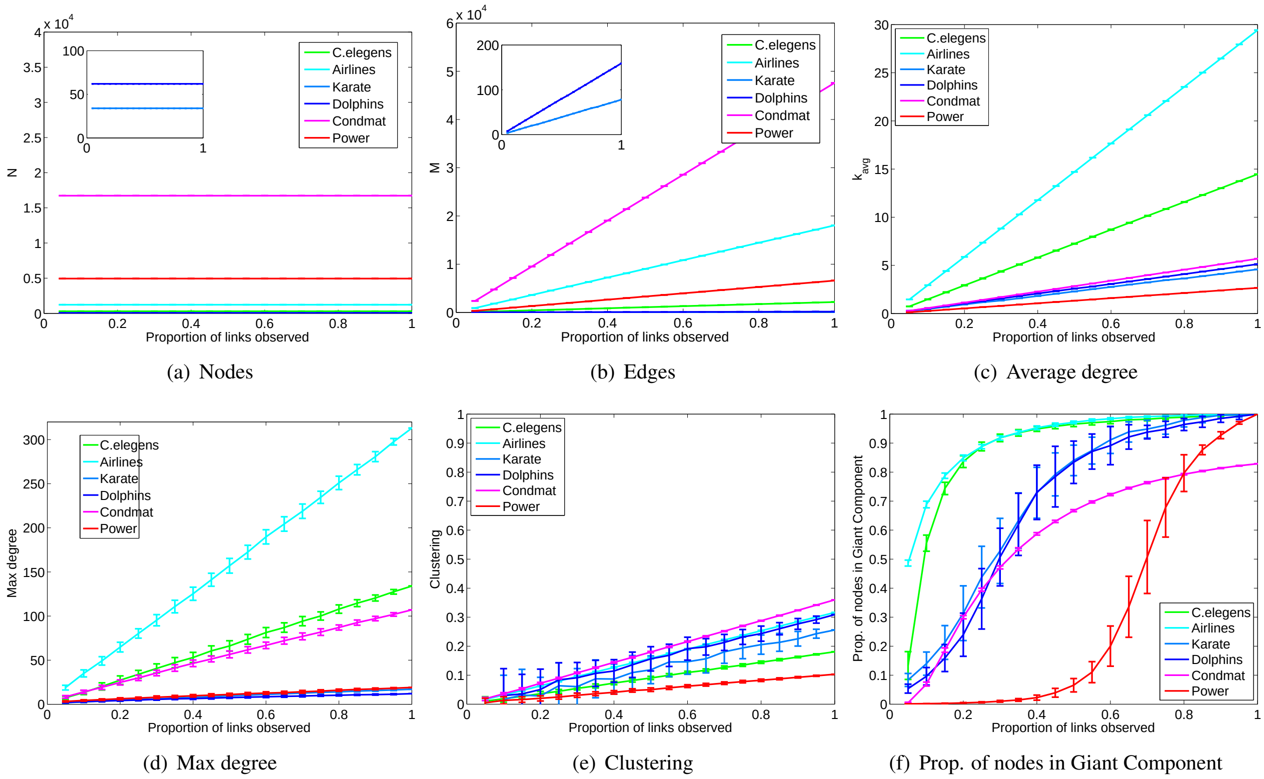

(a) Nodes (b) Edges (c) Average degree

(d) Max degree (e) Clustering (f) Prop. of nodes in Giant Component

Figure S6: Scaling of subnetwork statistics for empirical networks obtained by failing links. (a.) When all nodes are known q links are observed through sampling, the sample statistic for the number of nodes n equals the true number of nodes N. It should be noted, though, that some nodes of degree 0 may be observed and these are counted as nodes (not discarded). (b.) The number of edges scales linearly as $M_{\text{obs}} = qM$. (c.) The average degree scales linearly as $k_{\text{avg}}^{\text{obs}} = \frac{k_{\text{avg}}^{\text{true}}}{q}$. (d.) The max degree scales linearly (e.) Clustering scales roughly linearly with q. (f.) The percolation threshold roughly corresponds to the q for which $k_{\text{avg}} \geq 1$.

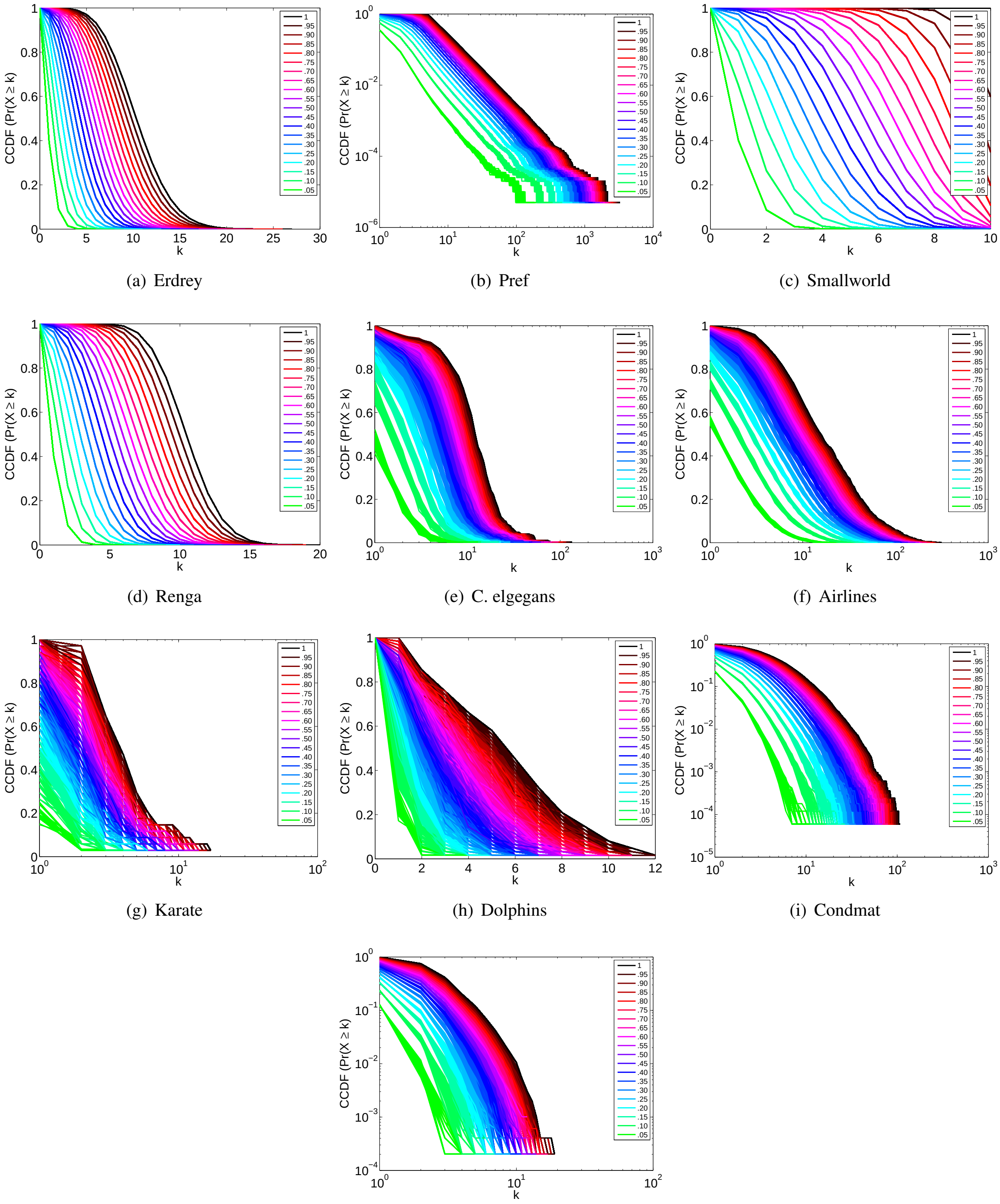

(a) Erdrey (b) Pref (c) Smallworld

(d) Renga (e) C. elgegans (f) Airlines

(g) Karate (h) Dolphins (i) Condmat

(j) Powergrid

Figure S7: CCDF distortion for subnetworks obtained by failing links. Subnetwork degree distributions do not capture the true degree distribution, especially for small q.

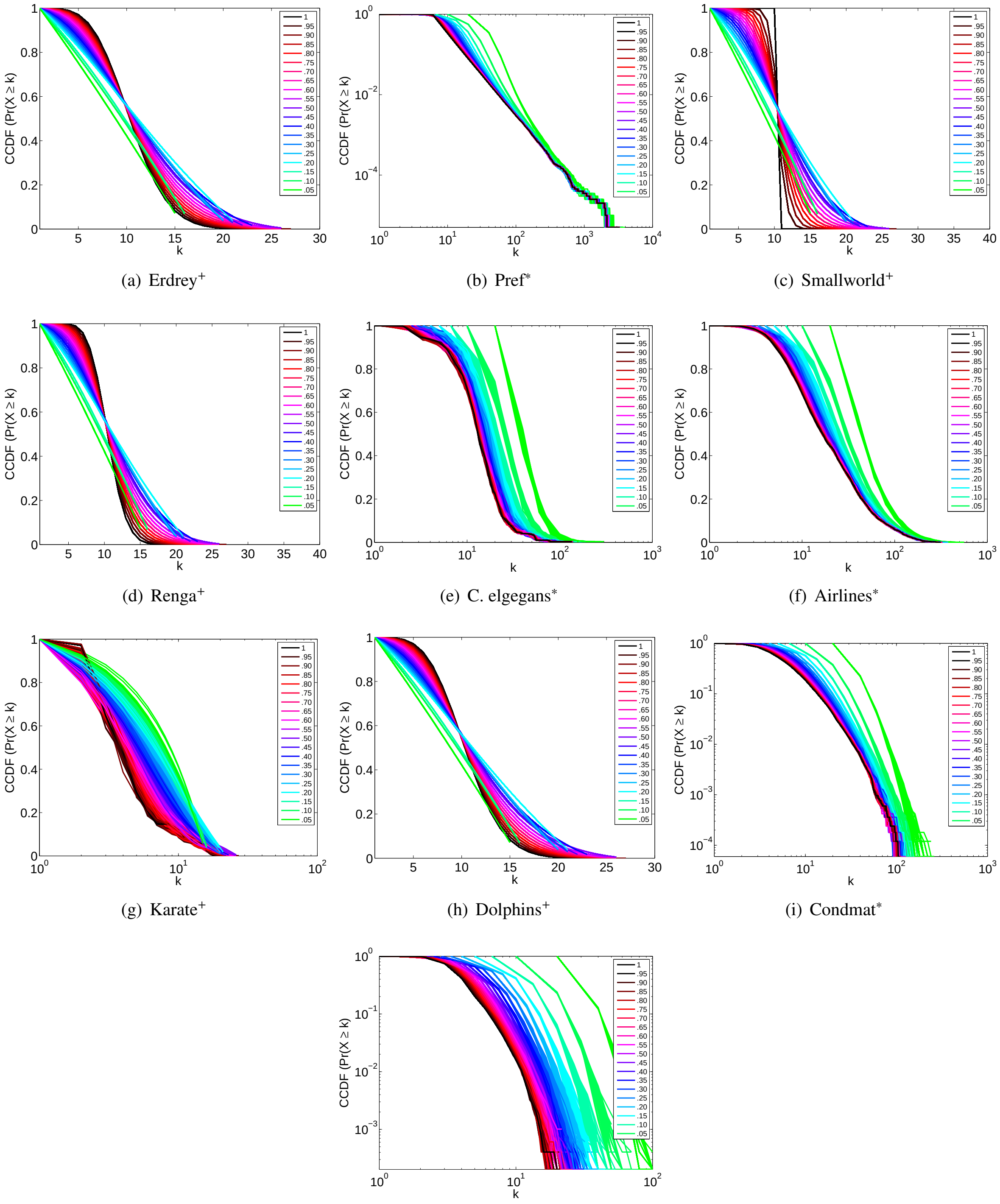

(a) Erdrey^{+} (b) Pref* (c) Smallworld^{+}

(d) Renga^{+} (e) C. elgegans* (f) Airlines*

(g) Karate^{+} (h) Dolphins^{+} (i) Condmat*

(j) Powergrid*

Figure S8: Predicted CCDF from subnetworks obtained by failing links. The predicted CCDF shows relatively good agreement with the CCDF for most networks. Karate club and Dolphins exhibit significant deviations, possibly due to the small number of nodes in these networks. Networks designated with $^{+}$ utilized Equation [16] and those designated with with * utilized Equation [18].

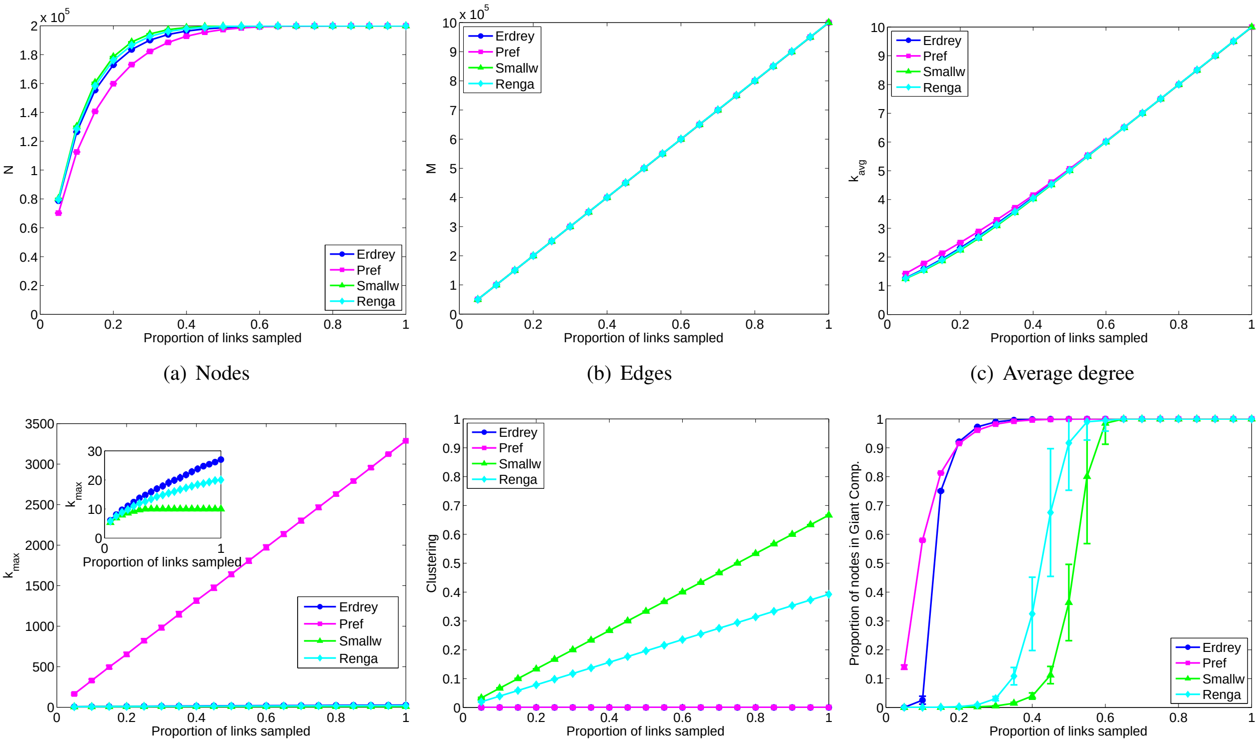

(a) Nodes (b) Edges (c) Average degree

(d) Max degree (e) Clustering (f) Prop. of links in Giant Component

Figure S9: Scaling of subnetwork statistics for simulated networks induced on sampled links. (a.) The number of nodes in a subnetwork sampled by links scales nonlinearly with q. (b.) The number of edges scales as $m \approx qM$. (c.) The average degree scales roughly linearly with the proportion of nodes subsampled $k_{\text{avg}}^{\text{sub}} \approx qk_{\text{avg}}$. (d.) The max degree scales roughly linearly for networks with few large hubs (e.g., Pref) and nonlinearly when there are several nodes with degrees roughly similar to k_{max}. (e.) The clustering coefficient scales roughly linearly $C^{\text{sub}} \approx qC$. (f.) The proportion of nodes in the giant component increases with the proportion of nodes sampled. For the random graphs (Erdrey and Pref) there is a critical point corresponding to the approximate sampling level when $k_{\text{avg}} > 1$ (which corresponds to $q = 0.1$). The thresholds for Small World and Range dependent networks are much higher due to the uniformity of the motif distribution in these networks. Markers indicates the mean over 100 simulations. Error bars showing one standard deviation are too small to see.

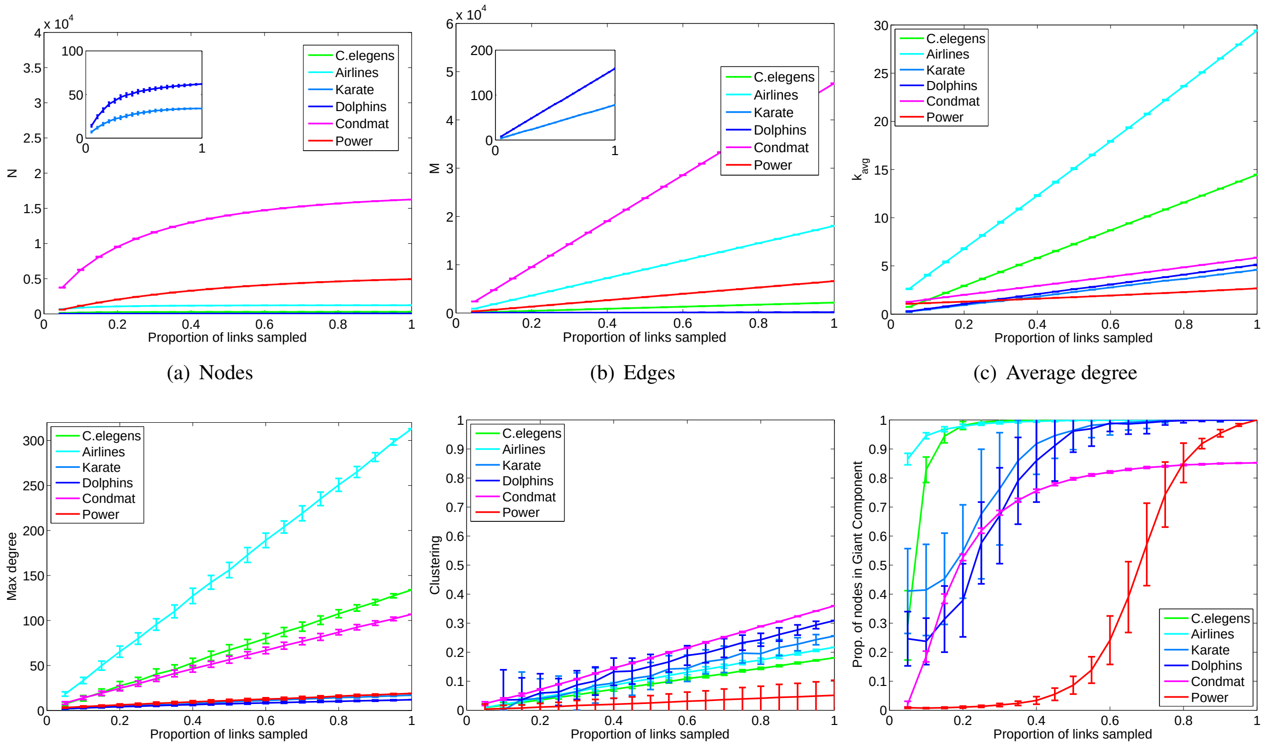

(a) Nodes (b) Edges (c) Average degree

(d) Max degree (e) Clustering (f) Giant Component

Figure S10: Scaling of subnetwork statistics for empirical networks induced on sampled links. (a.) The number of nodes in a subnetwork sampled by nodes scales nonlinearly with q. (b.) The number of edges scales as $m \approx qM$. (c.) The average degree scales roughly linearly with the proportion of nodes subsampled $k_{\text{avg}}^{\text{sub}} \approx qk_{\text{avg}}$. (d.) The max degree scales roughly linearly for networks with few large hubs. (e.) The clustering coefficient scales roughly linearly $C^{\text{sub}} \approx qC$. (f.) The proportion of nodes in the giant component increases with the proportion of links sampled. *C. elegans* and airlines maintain a large proportion of nodes in the giant component, most likely because these networks have high average degree. Karate club and dolphins show considerable variability (as shown by error bars ± s.d.) because these are relatively small networks. Powergrid is fragile to sampling by links, meaning the a high proportion of sampled links must be obtained to reach a fully connected network.

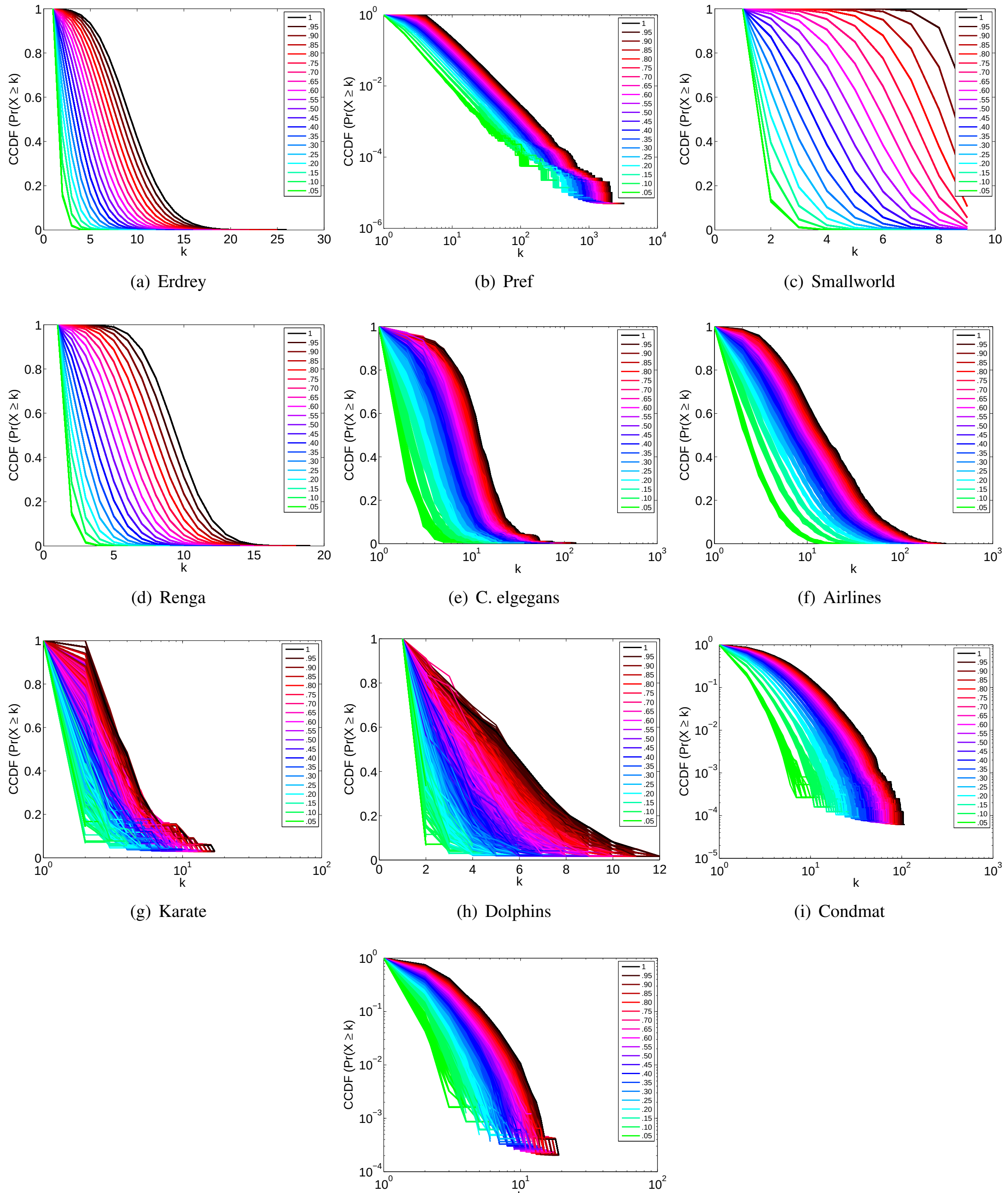

(a) Erdrey (b) Pref (c) Smallworld

(d) Renga (e) C. elgegans (f) Airlines

(g) Karate (h) Dolphins (i) Condmat

(j) Powergrid

Figure S11: CCDF distortion for subnetworks induced on sampled links. Subnetwork degree distributions do not capture the true degree distribution, especially for small q.

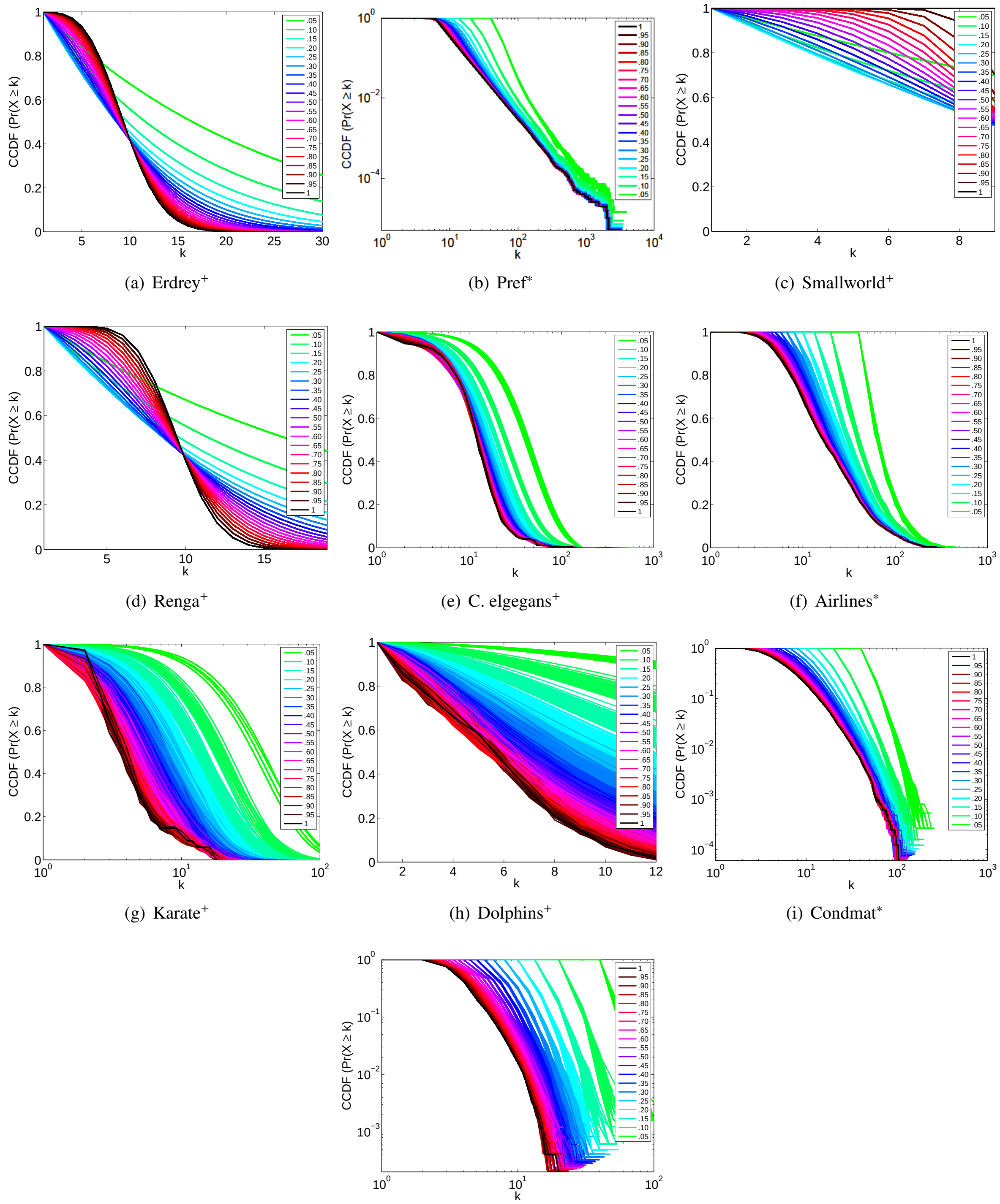

(a) Erdrey^{+} (b) Pref* (c) Smallworld^{+}

(d) Renga^{+} (e) C. elgegans^{+} (f) Airlines*

(g) Karate^{+} (h) Dolphins^{+} (i) Condmat*

(j) Powergrid*

Figure S12: Predicted CCDF from subnetworks induced on sampled links. The predicted CCDF shows relatively good agreement with the CCDF for most networks. Karate club and Dolphins exhibit significant deviations, possibly due to the small number of nodes in these networks. Networks designated with $^{+}$ utilized Equation [16] and those designated with with * utilized Equation [18].

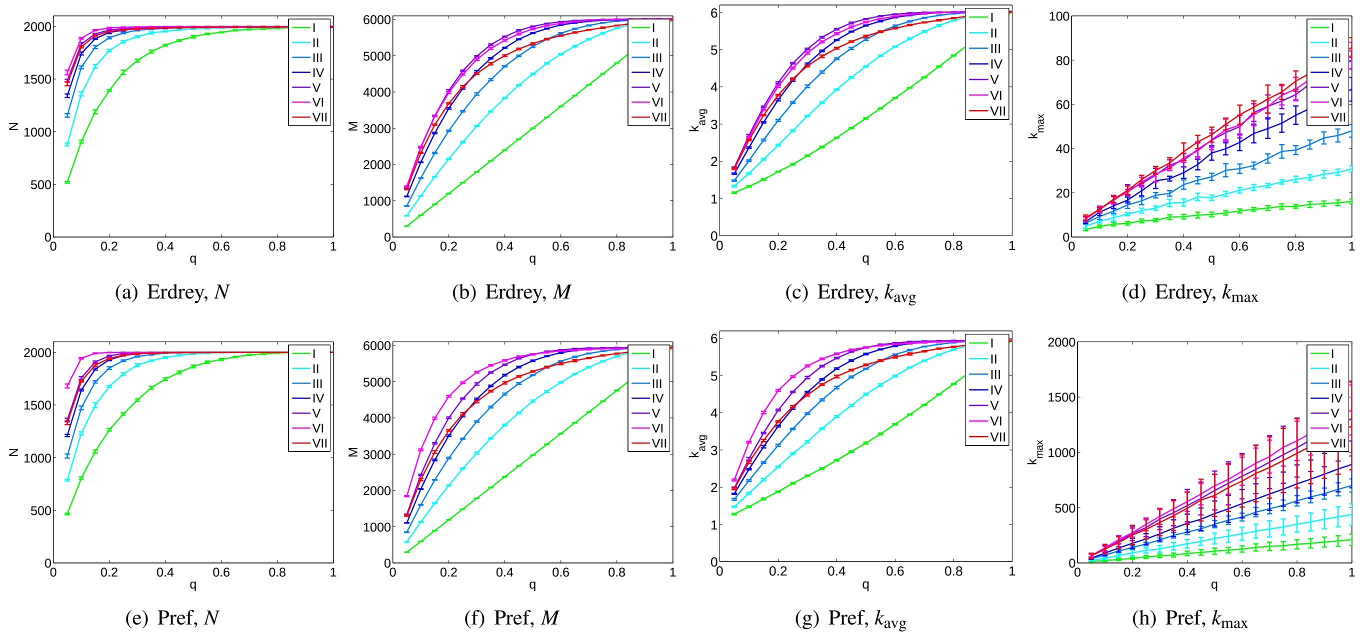

(a) Erdrey, N (b) Erdrey, M (c) Erdrey, k_{avg} (d) Erdrey, $k_{\max}$

(e) Pref, N (f) Pref, M (g) Pref, k_{avg} (h) Pref, $k_{\max}$

Figure S13: Scaling of subnetwork statistics for simulated networks induced on sampled interactions.

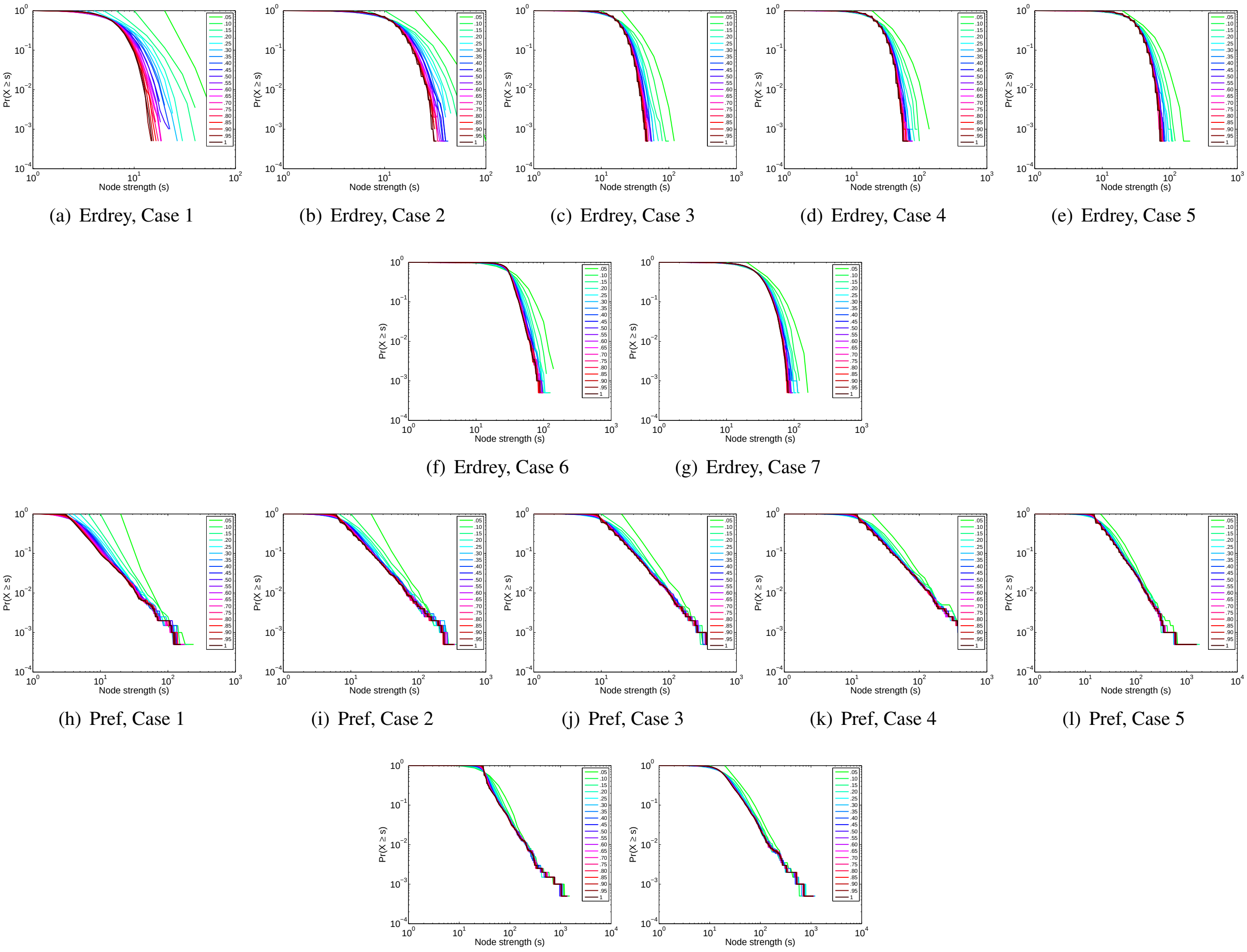

(a) Erdrey, Case 1 (b) Erdrey, Case 2 (c) Erdrey, Case 3 (d) Erdrey, Case 4 (e) Erdrey, Case 5

(f) Erdrey, Case 6 (g) Erdrey, Case 7

(h) Pref, Case 1 (i) Pref, Case 2 (j) Pref, Case 3 (k) Pref, Case 4 (l) Pref, Case 5

(m) Pref, Case 6 (n) Pref, Case 7

Figure S14: Predicted node strength distribution for weighted, simulated networks.

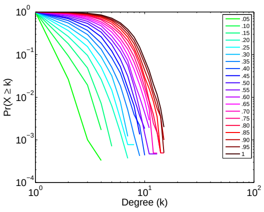

(a) Erdrey, Case 1

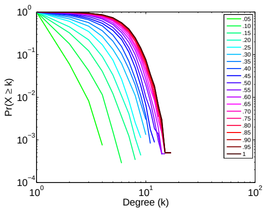

(b) Erdrey, Case 2

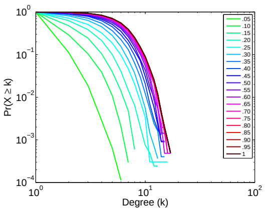

(c) Erdrey, Case 3

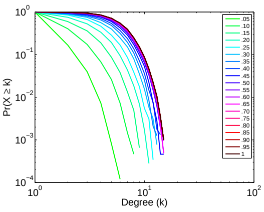

(d) Erdrey, Case 4

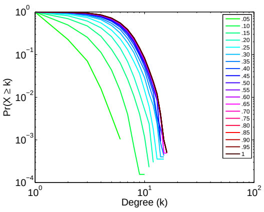

(e) Erdrey, Case 5

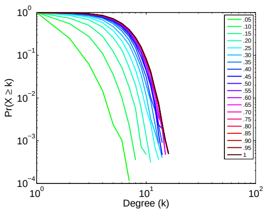

(f) Erdrey, Case 6

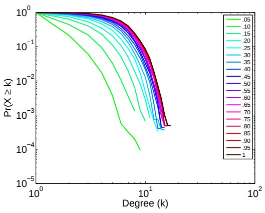

(g) Erdrey, Case 7

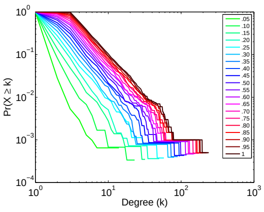

(h) Pref, Case 1

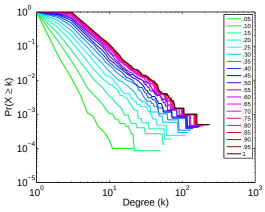

(i) Pref, Case 2

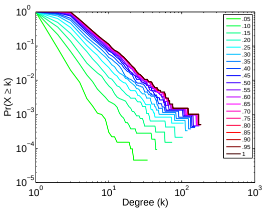

(j) Pref, Case 3

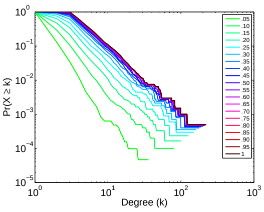

(k) Pref, Case 4

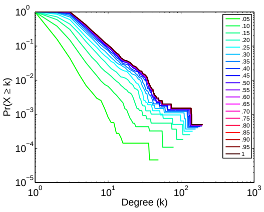

(l) Pref, Case 5

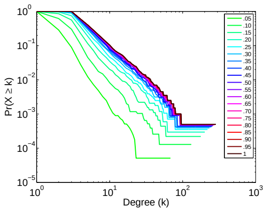

(m) Pref, Case 6

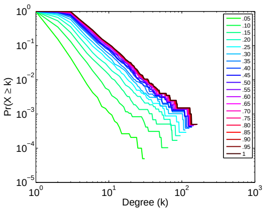

(n) Pref, Case 7

Figure S15: Predicted degree distribution for weighted, simulated networks.

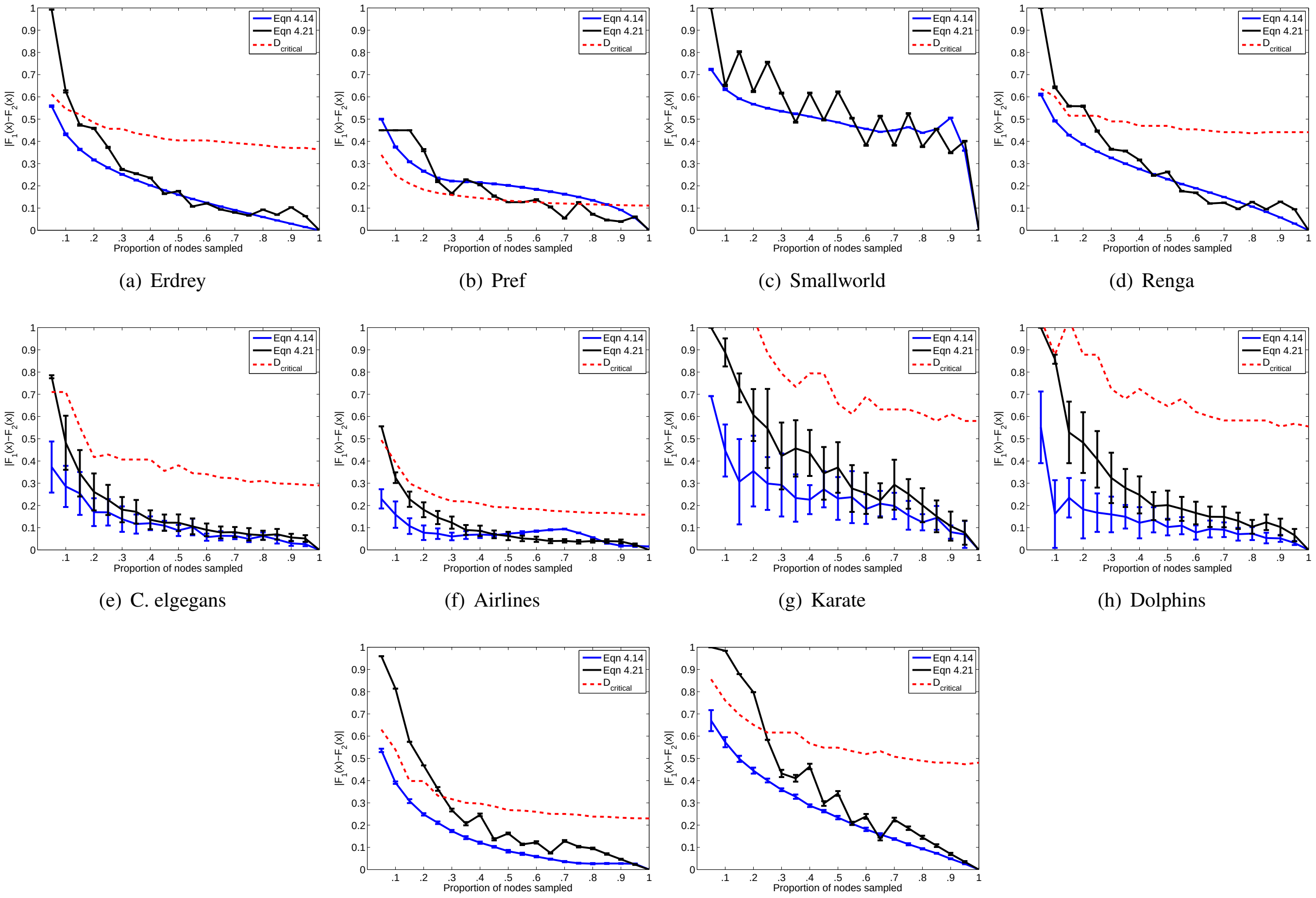

(a) Erdrey (b) Pref (c) Smallworld (d) Renga

(e) C. elgegans (f) Airlines (g) Karate (h) Dolphins

(i) Condmat (j) Powergrid

Figure S16: Kolmogorov-Smirnov two sample test for true CDF and predicted CDF from subnetworks induced on sampled nodes. The red line represents D_{crit} for $\alpha = 0.05$ and sample sizes $n_1 = k_{\text{max}}$ of the true CDF and $n_2 = k_{\text{max}}$ of the observed CDF. The predicted CDFs for for most networks are statistically indistinguishable from the true CDF for these networks for $q > 0.3$. Due to the presence of large hubs in Pref, n_1 and n_2 are quite large leading to high statistical power in the KS test. Thus, even very small differences between the true and predicted CDFs result in a statistically significant difference and rejection of the null hypothesis, even though the curves show relatively good agreement.

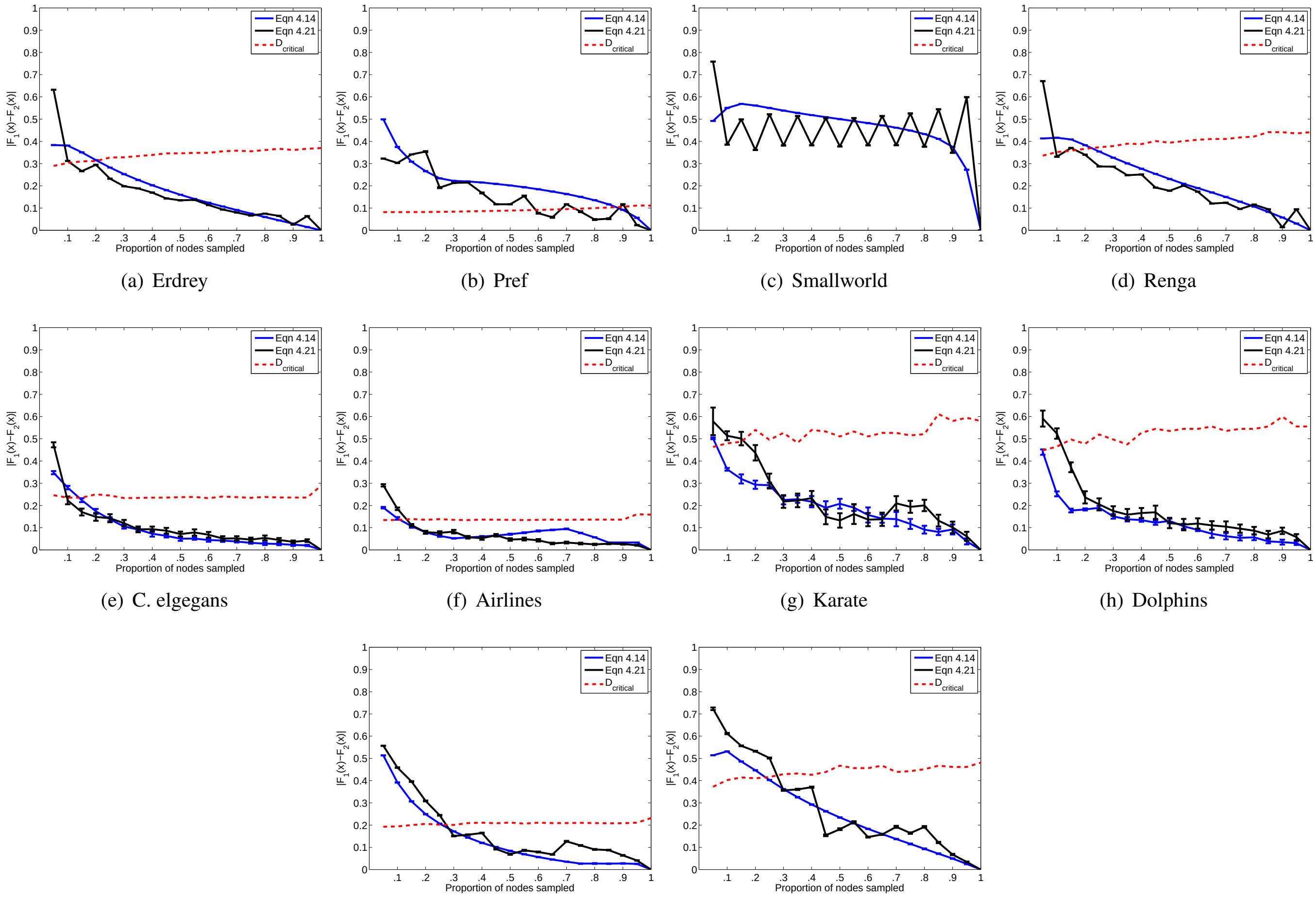

(a) Erdrey (b) Pref (c) Smallworld (d) Renga

(e) C. elgegans (f) Airlines (g) Karate (h) Dolphins

(i) Condmat (j) Powergrid

Figure S17: Kolmogorov-Smirnov two sample test for true CDF and predicted CDF from subnetworks obtained by failing links. The red line represents D_{crit} for $\alpha = 0.05$ and sample sizes $n_1 = k_{\text{max}}$ of the true CDF and $n_2 = k_{\text{max}}$ of the observed CDF. The predicted CDFs for for most networks are statistically indistinguishable from the true CDF for these networks for $q > 0.3$. Due to the presence of large hubs in Pref, n_1 and n_2 are quite large leading to high statistical power in the KS test. Thus, even very small differences between the true and predicted CDFs result in a statistically significant difference and rejection of the null hypothesis, even though the curves show relatively good agreement.

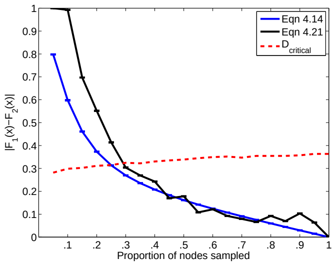

(a) Erdrey

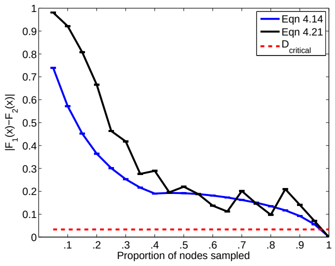

(b) Pref

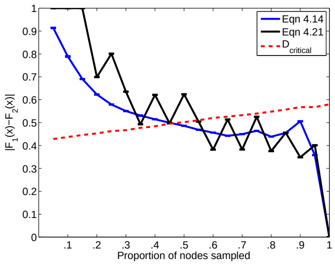

(c) Smallworld

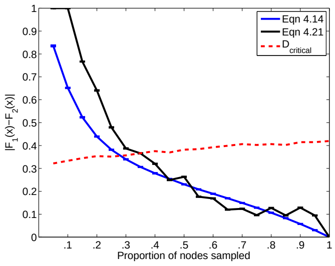

(d) Renga

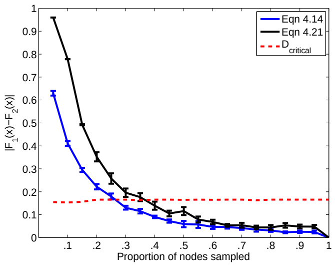

(e) C. elgegans

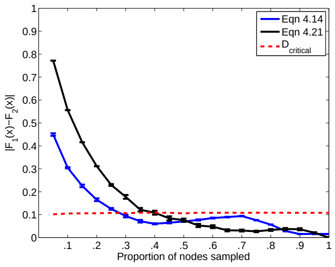

(f) Airlines

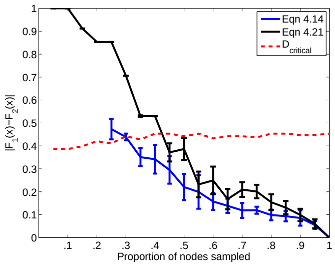

(g) Karate

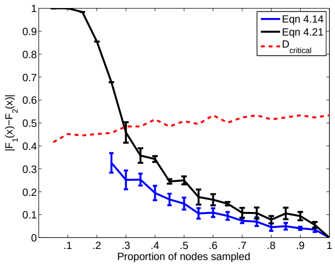

(h) Dolphins

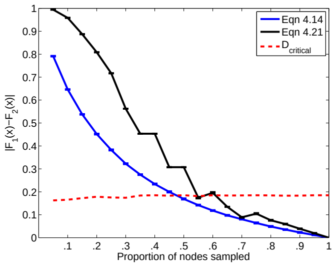

(i) Condmat

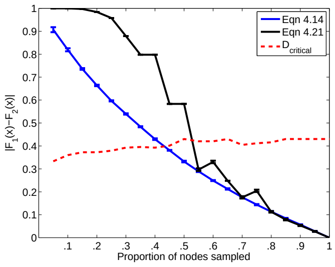

(j) Powergrid

Figure S18: Kolmogorov-Smirnov two sample test for true CDF and predicted CDF from subnetworks generated by sampled links. The red line represents D_{crit} for $\alpha = 0.05$ and sample sizes $n_1 = k_{\max}$ of the true CDF and $n_2 = k_{\max}$ of the observed CDF. The predicted CDFs for for most networks are statistically indistinguishable from the true CDF for these networks for $q > 0.3$. Due to the presence of large hubs in Pref, n_1 and n_2 are quite large leading to high statistical power in the KS test. Thus, even very small differences between the true and predicted CDFs result in a statistically significant difference and rejection of the null hypothesis, even though the curves show relatively good agreement.

Table S1: Error in $\hat{N}$ when sampling by nodes.

q	Erdrey	Pref	Smallw	Renga	C.elegans	Airlines	Karate	Dolphins	Condmat	Power
0.05	0.00	0.00	0.00	0.00	0.00	0.00	0.00	0.00	0.00	0.00
0.10	0.00	0.00	0.00	0.00	0.00	0.00	0.00	0.00	0.00	0.00
0.15	0.00	0.00	0.00	0.00	0.00	0.00	0.00	0.00	0.00	0.00
0.20	0.00	0.00	0.00	0.00	0.00	0.00	0.00	0.00	0.00	0.00
0.25	0.00	0.00	0.00	0.00	0.00	0.00	0.00	0.00	0.00	0.00
0.30	0.00	0.00	0.00	0.00	0.00	0.00	0.00	0.00	0.00	0.00
0.35	0.00	0.00	0.00	0.00	0.00	0.00	0.00	0.00	0.00	0.00
0.40	0.00	0.00	0.00	0.00	0.00	0.00	0.00	0.00	0.00	0.00
0.45	0.00	0.00	0.00	0.00	0.00	0.00	0.00	0.00	0.00	0.00
0.50	0.00	0.00	0.00	0.00	0.00	0.00	0.00	0.00	0.00	0.00
0.55	0.00	0.00	0.00	0.00	0.00	0.00	0.00	0.00	0.00	0.00
0.60	0.00	0.00	0.00	0.00	0.00	0.00	0.00	0.00	0.00	0.00
0.65	0.00	0.00	0.00	0.00	0.00	0.00	0.00	0.00	0.00	0.00
0.70	0.00	0.00	0.00	0.00	0.00	0.00	0.00	0.00	0.00	0.00
0.75	0.00	0.00	0.00	0.00	0.00	0.00	0.00	0.00	0.00	0.00
0.80	0.00	0.00	0.00	0.00	0.00	0.00	0.00	0.00	0.00	0.00
0.85	0.00	0.00	0.00	0.00	0.00	0.00	0.00	0.00	0.00	0.00
0.90	0.00	0.00	0.00	0.00	0.00	0.00	0.00	0.00	0.00	0.00
0.95	0.00	0.00	0.00	0.00	0.00	0.00	0.00	0.00	0.00	0.00
1.00	0.00	0.00	0.00	0.00	0.00	0.00	0.00	0.00	0.00	0.00

Table S2: Error in $\hat{M}$ when sampling by nodes. The percent error in the number of predicted nodes is nearly zero when, except in the small empirical networks where for small q, we violate the assumption that $n \gg 1$ and incur large errors.

q	Erdrey	Pref	Smallw	Renga	C. elegans	Airlines	Karate	Dolphins	Condmat	Power
0.05	0.00	0.00	0.00	0.00	0.08	0.02	2.71	2.04	0.00	0.01
0.10	0.00	0.00	0.00	0.00	0.02	0.03	1.04	0.28	0.00	0.00
0.15	0.00	0.00	0.00	0.00	0.01	0.00	0.24	0.04	0.00	0.01
0.20	0.00	0.00	0.00	0.00	0.01	0.01	0.03	0.02	0.00	0.00
0.25	0.00	0.00	0.00	0.00	0.06	0.01	0.08	0.06	0.01	0.00
0.30	0.00	0.00	0.00	0.00	0.02	0.03	0.05	0.02	0.00	0.00
0.35	0.00	0.00	0.00	0.00	0.02	0.01	0.02	0.04	0.00	0.00
0.40	0.00	0.00	0.00	0.00	0.00	0.01	0.01	0.01	0.00	0.00
0.45	0.00	0.00	0.00	0.00	0.01	0.01	0.04	0.03	0.00	0.00
0.50	0.00	0.00	0.00	0.00	0.00	0.00	0.07	0.01	0.00	0.00
0.55	0.00	0.00	0.00	0.00	0.00	0.00	0.05	0.03	0.00	0.00
0.60	0.00	0.00	0.00	0.00	0.00	0.02	0.01	0.01	0.00	0.00
0.65	0.00	0.00	0.00	0.00	0.01	0.00	0.01	0.00	0.00	0.00
0.70	0.00	0.00	0.00	0.00	0.01	0.01	0.00	0.02	0.00	0.00
0.75	0.00	0.00	0.00	0.00	0.00	0.01	0.00	0.01	0.00	0.00
0.80	0.00	0.00	0.00	0.00	0.01	0.00	0.02	0.01	0.00	0.00
0.85	0.00	0.00	0.00	0.00	0.00	0.00	0.00	0.00	0.00	0.00
0.90	0.00	0.00	0.00	0.00	0.00	0.00	0.00	0.01	0.00	0.00
0.95	0.00	0.00	0.00	0.00	0.00	0.00	0.00	0.00	0.00	0.00
1.00	0.00	0.00	0.00	0.00	0.00	0.00	0.00	0.00	0.00	0.00

Table S3: Error in $\hat{k}_{\text{avg}}$ when sampling by nodes. Errors in $\hat{M}$ are largely responsible for errors in $\hat{k}_{\text{avg}}$.

q	Erdrey	Pref	Smallw	Renga	C.elegans	Airlines	Karate	Dolphins	Condmat	Power
0.05	0.00	0.00	0.00	0.00	0.08	0.02	2.71	2.04	0.00	0.01
0.10	0.00	0.00	0.00	0.00	0.02	0.03	1.04	0.28	0.00	0.00
0.15	0.00	0.00	0.00	0.00	0.01	0.00	0.24	0.04	0.00	0.01
0.20	0.00	0.00	0.00	0.00	0.01	0.01	0.03	0.02	0.00	0.00
0.25	0.00	0.00	0.00	0.00	0.06	0.01	0.08	0.06	0.01	0.00
0.30	0.00	0.00	0.00	0.00	0.02	0.03	0.05	0.02	0.00	0.00
0.35	0.00	0.00	0.00	0.00	0.02	0.01	0.02	0.04	0.00	0.00
0.40	0.00	0.00	0.00	0.00	0.00	0.01	0.01	0.01	0.00	0.00
0.45	0.00	0.00	0.00	0.00	0.01	0.01	0.04	0.03	0.00	0.00
0.50	0.00	0.00	0.00	0.00	0.00	0.00	0.07	0.01	0.00	0.00
0.55	0.00	0.00	0.00	0.00	0.00	0.00	0.05	0.03	0.00	0.00
0.60	0.00	0.00	0.00	0.00	0.00	0.02	0.01	0.01	0.00	0.00
0.65	0.00	0.00	0.00	0.00	0.01	0.00	0.01	0.00	0.00	0.00
0.70	0.00	0.00	0.00	0.00	0.01	0.01	0.00	0.02	0.00	0.00
0.75	0.00	0.00	0.00	0.00	0.00	0.01	0.00	0.01	0.00	0.00
0.80	0.00	0.00	0.00	0.00	0.01	0.00	0.02	0.01	0.00	0.00
0.85	0.00	0.00	0.00	0.00	0.00	0.00	0.00	0.00	0.00	0.00
0.90	0.00	0.00	0.00	0.00	0.00	0.00	0.00	0.01	0.00	0.00
0.95	0.00	0.00	0.00	0.00	0.00	0.00	0.00	0.00	0.00	0.00
1.00	0.00	0.00	0.00	0.00	0.00	0.00	0.00	0.00	0.00	0.00

Table S4: Error in $\hat{k}_{\text{max}}$ when sampling by nodes. The percent error in the predicted max degree is nearly zero for large q. In general, predicting the max. degree is difficult due to the dependence on network structure.

q	Erdrey	Pref	Smallw	Renga	C. elegans	Airlines	Karate	Dolphins	Condmat	Power
0.05	2.70	0.67	7.70	3.73	0.59	0.39	0.00	0.08	0.13	0.14
0.10	1.60	0.54	4.94	2.26	0.52	0.28	0.18	0.02	0.09	0.08
0.15	1.13	0.49	3.73	1.67	0.46	0.21	0.31	0.01	0.05	0.05
0.20	0.89	0.42	2.96	1.29	0.46	0.18	0.30	0.01	0.06	0.04
0.25	0.72	0.38	2.46	1.06	0.44	0.15	0.28	0.01	0.05	0.03
0.30	0.57	0.33	2.09	0.87	0.33	0.12	0.21	0.01	0.05	0.02
0.35	0.48	0.27	1.77	0.73	0.33	0.12	0.18	0.01	0.06	0.01
0.40	0.40	0.24	1.50	0.62	0.30	0.09	0.10	0.01	0.05	0.01
0.45	0.34	0.21	1.22	0.52	0.25	0.07	0.17	0.01	0.02	0.01
0.50	0.29	0.19	1.00	0.44	0.20	0.07	0.13	0.01	0.01	0.01
0.55	0.24	0.16	0.82	0.38	0.20	0.07	0.11	0.01	0.01	0.01
0.60	0.21	0.15	0.67	0.33	0.19	0.04	0.04	0.01	0.00	0.01
0.65	0.17	0.13	0.54	0.27	0.16	0.04	0.03	0.01	0.00	0.00
0.70	0.14	0.10	0.43	0.23	0.10	0.04	0.02	0.00	0.01	0.00
0.75	0.11	0.07	0.33	0.18	0.12	0.04	0.01	0.00	0.01	0.00
0.80	0.09	0.05	0.25	0.13	0.10	0.02	0.01	0.00	0.01	0.00
0.85	0.07	0.04	0.18	0.10	0.07	0.01	0.01	0.00	0.00	0.00
0.90	0.04	0.03	0.11	0.07	0.04	0.01	0.00	0.00	0.00	0.00
0.95	0.02	0.02	0.05	0.04	0.03	0.01	0.01	0.00	0.00	0.00
1.00	0.00	0.00	0.00	0.00	0.00	0.00	0.00	0.00	0.00	0.00

Table S5: Error in $\hat{C}$ when sampling by nodes. For some small networks with a small portion of nodes sampled q, no paths of length three occurred and the clustering coefficient was not computed in these cases.

q	Erdrey	Pref	Smallw	Renga	C. elegans	Airlines	Karate	Dolphins	Condmat	Power
0.05	0.01	0.33	0.00	0.00	–	0.04	–	–	0.00	–
0.10	0.07	0.15	0.00	0.00	0.09	0.02	–	–	0.01	0.21
0.15	0.04	0.10	0.00	0.00	0.08	0.02	–	–	0.01	0.03
0.20	0.03	0.07	0.00	0.00	0.01	0.02	–	–	0.01	0.02
0.25	0.05	0.06	0.00	0.00	0.01	0.01	–	–	0.00	0.07
0.30	0.04	0.05	0.00	0.00	0.05	0.01	–	0.15	0.00	0.03
0.35	0.05	0.03	0.00	0.00	0.03	0.01	–	0.14	0.00	0.06
0.40	0.05	0.02	0.00	0.00	0.00	0.01	0.06	0.12	0.00	0.02
0.45	0.03	0.02	0.00	0.00	0.01	0.01	0.13	0.02	0.00	0.05
0.50	0.01	0.02	0.00	0.00	0.01	0.01	0.20	0.04	0.00	0.02
0.55	0.00	0.01	0.00	0.00	0.01	0.00	0.12	0.05	0.00	0.00
0.60	0.02	0.00	0.00	0.00	0.01	0.00	0.08	0.02	0.00	0.02
0.65	0.01	0.00	0.00	0.00	0.01	0.00	0.04	0.01	0.00	0.01
0.70	0.01	0.00	0.00	0.00	0.00	0.00	0.04	0.01	0.00	0.00
0.75	0.00	0.01	0.00	0.00	0.01	0.00	0.03	0.00	0.00	0.00
0.80	0.00	0.01	0.00	0.00	0.00	0.00	0.01	0.00	0.00	0.00
0.85	0.00	0.01	0.00	0.00	0.00	0.00	0.02	0.01	0.00	0.00
0.90	0.00	0.01	0.00	0.00	0.00	0.00	0.01	0.01	0.00	0.01
0.95	0.00	0.00	0.00	0.00	0.00	0.00	0.01	0.00	0.00	0.00
1.00	0.00	0.00	0.00	0.00	0.00	0.00	0.00	0.00	0.00	0.00

Table S6: Error in $\hat{N}$ when sampling by failing links. No error is encountered because all nodes remain in the subnetwork.

q	Erdrey	Pref	Smallw	Renga	C. elegans	Airlines	Karate	Dolphins	Condmat	Power
0.05	0.00	0.00	0.00	0.00	0.00	0.00	0.00	0.00	0.00	0.00
0.10	0.00	0.00	0.00	0.00	0.00	0.00	0.00	0.00	0.00	0.00
0.15	0.00	0.00	0.00	0.00	0.00	0.00	0.00	0.00	0.00	0.00
0.20	0.00	0.00	0.00	0.00	0.00	0.00	0.00	0.00	0.00	0.00
0.25	0.00	0.00	0.00	0.00	0.00	0.00	0.00	0.00	0.00	0.00
0.30	0.00	0.00	0.00	0.00	0.00	0.00	0.00	0.00	0.00	0.00
0.35	0.00	0.00	0.00	0.00	0.00	0.00	0.00	0.00	0.00	0.00
0.40	0.00	0.00	0.00	0.00	0.00	0.00	0.00	0.00	0.00	0.00
0.45	0.00	0.00	0.00	0.00	0.00	0.00	0.00	0.00	0.00	0.00
0.50	0.00	0.00	0.00	0.00	0.00	0.00	0.00	0.00	0.00	0.00
0.55	0.00	0.00	0.00	0.00	0.00	0.00	0.00	0.00	0.00	0.00
0.60	0.00	0.00	0.00	0.00	0.00	0.00	0.00	0.00	0.00	0.00
0.65	0.00	0.00	0.00	0.00	0.00	0.00	0.00	0.00	0.00	0.00
0.70	0.00	0.00	0.00	0.00	0.00	0.00	0.00	0.00	0.00	0.00
0.75	0.00	0.00	0.00	0.00	0.00	0.00	0.00	0.00	0.00	0.00
0.80	0.00	0.00	0.00	0.00	0.00	0.00	0.00	0.00	0.00	0.00
0.85	0.00	0.00	0.00	0.00	0.00	0.00	0.00	0.00	0.00	0.00
0.90	0.00	0.00	0.00	0.00	0.00	0.00	0.00	0.00	0.00	0.00
0.95	0.00	0.00	0.00	0.00	0.00	0.00	0.00	0.00	0.00	0.00
1.00	0.00	0.00	0.00	0.00	0.00	0.00	0.00	0.00	0.00	0.00

Table S7: Error in $\hat{M}$ when sampling by failing links. Since we are sampling qM links, errors in predicting the true number of links are quite small and nonzero only due to roundoff error (e.g., m =round(qM)).

q	Erdrey	Pref	Smallw	Renga	C. elegans	Airlines	Karate	Dolphins	Condmat	Power
0.05	0.00	0.00	0.00	0.00	0.00	0.00	0.03	0.01	0.00	0.00
0.10	0.00	0.00	0.00	0.00	0.00	0.00	0.03	0.01	0.00	0.00
0.15	0.00	0.00	0.00	0.00	0.00	0.00	0.03	0.01	0.00	0.00
0.20	0.00	0.00	0.00	0.00	0.00	0.00	0.03	0.01	0.00	0.00
0.25	0.00	0.00	0.00	0.00	0.00	0.00	0.03	0.01	0.00	0.00
0.30	0.00	0.00	0.00	0.00	0.00	0.00	0.02	0.01	0.00	0.00
0.35	0.00	0.00	0.00	0.00	0.00	0.00	0.01	0.01	0.00	0.00
0.40	0.00	0.00	0.00	0.00	0.00	0.00	0.01	0.01	0.00	0.00
0.45	0.00	0.00	0.00	0.00	0.00	0.00	0.00	0.01	0.00	0.00
0.50	0.00	0.00	0.00	0.00	0.00	0.00	0.00	0.01	0.00	0.00
0.55	0.00	0.00	0.00	0.00	0.00	0.00	0.00	0.01	0.00	0.00
0.60	0.00	0.00	0.00	0.00	0.00	0.00	0.00	0.00	0.00	0.00
0.65	0.00	0.00	0.00	0.00	0.00	0.00	0.01	0.00	0.00	0.00
0.70	0.00	0.00	0.00	0.00	0.00	0.00	0.01	0.00	0.00	0.00
0.75	0.00	0.00	0.00	0.00	0.00	0.00	0.01	0.00	0.00	0.00
0.80	0.00	0.00	0.00	0.00	0.00	0.00	0.01	0.00	0.00	0.00
0.85	0.00	0.00	0.00	0.00	0.00	0.00	0.00	0.00	0.00	0.00
0.90	0.00	0.00	0.00	0.00	0.00	0.00	0.00	0.00	0.00	0.00
0.95	0.00	0.00	0.00	0.00	0.00	0.00	0.00	0.00	0.00	0.00
1.00	0.00	0.00	0.00	0.00	0.00	0.00	0.00	0.00	0.00	0.00

Table S8: Error in $\hat{k}_{\rm avg}$ when sampling by failing links. The predicted average degree is computed from $\hat{N}$ and $\hat{M}$. Error in the predicted average agree are small and only occur due to rounding errors in the selecting an integer number of qM edges in the random sample.

q	Erdrey	Pref	Smallw	Renga	C. elegans	Airlines	Karate	Dolphins	Condmat	Power
0.05	0.00	0.00	0.00	0.00	0.03	0.03	0.06	0.09	0.00	0.00
0.10	0.00	0.00	0.00	0.00	0.02	0.02	0.03	0.05	0.00	0.00
0.15	0.00	0.00	0.00	0.00	0.02	0.01	0.03	0.03	0.00	0.00
0.20	0.00	0.00	0.00	0.00	0.01	0.01	0.03	0.02	0.00	0.00
0.25	0.00	0.00	0.00	0.00	0.01	0.01	0.03	0.02	0.00	0.00
0.30	0.00	0.00	0.00	0.00	0.01	0.01	0.02	0.02	0.00	0.00
0.35	0.00	0.00	0.00	0.00	0.01	0.00	0.01	0.01	0.00	0.00
0.40	0.00	0.00	0.00	0.00	0.00	0.00	0.01	0.01	0.00	0.00
0.45	0.00	0.00	0.00	0.00	0.00	0.00	0.00	0.01	0.00	0.00
0.50	0.00	0.00	0.00	0.00	0.00	0.00	0.00	0.01	0.00	0.00
0.55	0.00	0.00	0.00	0.00	0.00	0.00	0.00	0.00	0.00	0.00
0.60	0.00	0.00	0.00	0.00	0.00	0.00	0.00	0.00	0.00	0.00
0.65	0.00	0.00	0.00	0.00	0.00	0.00	0.01	0.00	0.00	0.00
0.70	0.00	0.00	0.00	0.00	0.00	0.00	0.01	0.00	0.00	0.00
0.75	0.00	0.00	0.00	0.00	0.00	0.00	0.01	0.00	0.00	0.00
0.80	0.00	0.00	0.00	0.00	0.00	0.00	0.01	0.00	0.00	0.00
0.85	0.00	0.00	0.00	0.00	0.00	0.00	0.00	0.00	0.00	0.00
0.90	0.00	0.00	0.00	0.00	0.00	0.00	0.00	0.00	0.00	0.00
0.95	0.00	0.00	0.00	0.00	0.00	0.00	0.00	0.00	0.00	0.00
1.00	0.00	0.00	0.00	0.00	0.00	0.00	0.00	0.00	0.00	0.00

Table S9: Error in $\hat{C}$ when sampling by failing links.

q	Erdrey	Pref	Smallw	Renga	C. elegans	Airlines	Karate	Dolphins	Condmat	Power
0.05	0.50	0.11	0.00	0.01	0.13	0.18	–	–	0.06	0.28
0.10	0.66	0.05	0.00	0.00	0.05	0.03	0.36	0.04	0.02	0.18
0.15	0.18	0.02	0.00	0.00	0.00	0.01	0.18	0.24	0.06	0.05
0.20	0.06	0.02	0.00	0.00	0.00	0.04	0.45	0.18	0.03	0.01
0.25	0.05	0.00	0.00	0.00	0.02	0.00	0.01	0.09	0.10	0.04
0.30	0.03	0.01	0.00	0.00	0.01	0.00	0.21	0.02	0.03	0.00
0.35	0.01	0.01	0.00	0.00	0.01	0.01	0.02	0.02	0.01	0.02
0.40	0.02	0.01	0.00	0.00	0.01	0.01	0.15	0.07	0.00	0.01
0.45	0.01	0.01	0.00	0.00	0.01	0.01	0.05	0.02	0.04	0.00
0.50	0.01	0.00	0.00	0.00	0.00	0.01	0.07	0.01	0.02	0.01
0.55	0.01	0.00	0.00	0.00	0.00	0.00	0.03	0.01	0.00	0.01
0.60	0.00	0.00	0.00	0.00	0.01	0.00	0.05	0.03	0.00	0.00
0.65	0.00	0.01	0.00	0.00	0.00	0.01	0.06	0.02	0.00	0.00
0.70	0.00	0.00	0.00	0.00	0.00	0.00	0.01	0.02	0.01	0.00
0.75	0.01	0.00	0.00	0.00	0.01	0.01	0.01	0.00	0.01	0.00
0.80	0.01	0.00	0.00	0.00	0.00	0.01	0.00	0.02	0.00	0.00
0.85	0.00	0.00	0.00	0.00	0.00	0.00	0.02	0.01	0.00	0.00
0.90	0.00	0.00	0.00	0.00	0.00	0.00	0.02	0.00	0.01	0.00
0.95	0.01	0.00	0.00	0.00	0.00	0.00	0.00	0.01	0.00	0.00
1.00	0.00	0.00	0.00	0.00	0.00	0.00	0.00	0.00	0.00	0.00

Table S10: Error in $\hat{k}_{\max}$ when sampling by failing links.

q	Erdrey	Pref	Smallw	Renga	C. elegans	Airlines	Karate	Dolphins	Condmat	Power
0.05	0.47	0.01	0.33	0.23	0.07	0.17	0.21	0.18	0.06	0.24
0.10	0.29	0.00	0.02	0.39	0.02	0.17	0.05	0.13	0.15	0.37
0.15	0.26	0.00	0.11	0.32	0.01	0.13	0.17	0.10	0.12	0.19
0.20	0.26	0.00	0.09	0.32	0.01	0.09	0.08	0.00	0.12	0.07
0.25	0.09	0.01	0.09	0.33	0.01	0.05	0.11	0.11	0.08	0.13
0.30	0.24	0.00	0.02	0.26	0.01	0.06	0.03	0.07	0.09	0.01
0.35	0.21	0.00	0.00	0.21	0.01	0.03	0.10	0.10	0.08	0.02
0.40	0.09	0.00	0.00	0.18	0.01	0.05	0.08	0.02	0.07	0.01
0.45	0.09	0.00	0.00	0.19	0.00	0.04	0.07	0.06	0.07	0.01
0.50	0.00	0.00	0.00	0.13	0.01	0.04	0.13	0.05	0.05	0.04
0.55	0.06	0.00	0.00	0.09	0.00	0.01	0.10	0.02	0.05	0.03
0.60	0.06	0.00	0.00	0.12	0.01	0.02	0.08	0.04	0.03	0.05
0.65	0.02	0.00	0.00	0.14	0.00	0.03	0.08	0.03	0.03	0.01
0.70	0.02	0.00	0.00	0.11	0.00	0.02	0.06	0.02	0.02	0.02
0.75	0.05	0.00	0.00	0.09	0.00	0.02	0.06	0.03	0.01	0.02
0.80	0.00	0.00	0.00	0.06	0.01	0.01	0.04	0.02	0.02	0.04
0.85	0.02	0.00	0.00	0.04	0.01	0.00	0.03	0.02	0.02	0.04
0.90	0.00	0.00	0.06	0.03	0.00	0.00	0.02	0.01	0.01	0.02
0.95	0.00	0.00	0.05	0.01	0.00	0.00	0.01	0.01	0.00	0.01
1.00	0.00	0.00	0.00	0.00	0.00	0.00	0.00	0.09	0.00	0.00

Table S11: Error in $\hat{N}$ when sampling by links. Predictors show good agreements with true values, except for low values of q. In these cases, errors in the predicted degree distribution contribute to errors in the predicted number of nodes. Future improvements in the predicted degree distribution would improve $\hat{N}$.

q	Erdrey	Pref	Smallw	Renga	C. elegans	Airlines	Karate	Dolphins	Condmat	Power
0.05	0.40	0.47	0.38	0.39	0.34	0.53	0.68	0.64	0.65	0.80
0.10	0.11	0.21	0.08	0.09	0.11	0.34	0.46	0.41	0.44	0.64
0.15	0.02	0.06	0.06	0.04	0.02	0.23	0.33	0.26	0.31	0.51
0.20	0.07	0.02	0.10	0.09	0.01	0.17	0.23	0.16	0.22	0.40
0.25	0.08	0.05	0.10	0.09	0.01	0.12	0.15	0.10	0.15	0.31
0.30	0.07	0.07	0.08	0.08	0.01	0.10	0.10	0.06	0.11	0.24
0.35	0.05	0.07	0.06	0.06	0.01	0.07	0.06	0.04	0.07	0.18
0.40	0.04	0.06	0.04	0.04	0.00	0.06	0.04	0.03	0.05	0.14
0.45	0.03	0.05	0.02	0.03	0.00	0.04	0.00	0.02	0.03	0.10
0.50	0.02	0.04	0.01	0.02	0.00	0.03	0.01	0.02	0.02	0.07
0.55	0.01	0.03	0.01	0.01	0.00	0.03	0.01	0.01	0.01	0.05
0.60	0.01	0.02	0.00	0.00	0.00	0.01	0.02	0.01	0.01	0.03
0.65	0.00	0.01	0.00	0.00	0.00	0.01	0.02	0.01	0.00	0.02
0.70	0.00	0.01	0.00	0.00	0.00	0.00	0.02	0.00	0.00	0.01
0.75	0.00	0.00	0.00	0.00	0.00	0.01	0.02	0.00	0.00	0.00
0.80	0.00	0.00	0.00	0.00	0.00	0.00	0.01	0.00	0.00	0.00
0.85	0.00	0.00	0.00	0.00	0.00	0.00	0.01	0.00	0.00	0.00
0.90	0.00	0.00	0.00	0.00	0.00	0.00	0.00	0.00	0.00	0.00
0.95	0.00	0.00	0.00	0.00	0.00	0.00	0.00	0.00	0.00	0.00
1.00	0.00	0.00	0.00	0.00	0.00	0.00	0.00	0.00	0.00	0.00

Table S12: Error in $\hat{M}$ when sampling by links. Error is nonzero only because of roundoff errors when selecting an integer number of edges to sample.

q	Erdrey	Pref	Smallw	Renga	C. elegans	Airlines	Karate	Dolphins	Condmat	Power
0.05	0.00	0.00	0.00	0.00	0.00	0.00	0.03	0.01	0.00	0.00
0.10	0.00	0.00	0.00	0.00	0.00	0.00	0.03	0.01	0.00	0.00
0.15	0.00	0.00	0.00	0.00	0.00	0.00	0.03	0.01	0.00	0.00
0.20	0.00	0.00	0.00	0.00	0.00	0.00	0.03	0.01	0.00	0.00
0.25	0.00	0.00	0.00	0.00	0.00	0.00	0.03	0.01	0.00	0.00
0.30	0.00	0.00	0.00	0.00	0.00	0.00	0.02	0.01	0.00	0.00
0.35	0.00	0.00	0.00	0.00	0.00	0.00	0.01	0.01	0.00	0.00
0.40	0.00	0.00	0.00	0.00	0.00	0.00	0.01	0.01	0.00	0.00
0.45	0.00	0.00	0.00	0.00	0.00	0.00	0.00	0.01	0.00	0.00
0.50	0.00	0.00	0.00	0.00	0.00	0.00	0.00	0.01	0.00	0.00
0.55	0.00	0.00	0.00	0.00	0.00	0.00	0.00	0.01	0.00	0.00
0.60	0.00	0.00	0.00	0.00	0.00	0.00	0.00	0.00	0.00	0.00
0.65	0.00	0.00	0.00	0.00	0.00	0.00	0.01	0.00	0.00	0.00
0.70	0.00	0.00	0.00	0.00	0.00	0.00	0.01	0.00	0.00	0.00
0.75	0.00	0.00	0.00	0.00	0.00	0.00	0.01	0.00	0.00	0.00
0.80	0.00	0.00	0.00	0.00	0.00	0.00	0.01	0.00	0.00	0.00
0.85	0.00	0.00	0.00	0.00	0.00	0.00	0.00	0.00	0.00	0.00
0.90	0.00	0.00	0.00	0.00	0.00	0.00	0.00	0.00	0.00	0.00
0.95	0.00	0.00	0.00	0.00	0.00	0.00	0.00	0.00	0.00	0.00
1.00	0.00	0.00	0.00	0.00	0.00	0.00	0.00	0.00	0.00	0.00

Table S13: Error in $\hat{k}_{\text{avg}}$ when sampling by links.

q	Erdrey	Pref	Smallw	Renga	C. elegans	Airlines	Karate	Dolphins	Condmat	Power
0.05	0.66	0.89	0.61	0.63	0.50	1.13	2.18	1.79	1.83	4.03
0.10	0.12	0.26	0.08	0.10	0.12	0.51	0.90	0.71	0.79	1.79
0.15	0.02	0.07	0.05	0.04	0.02	0.30	0.52	0.36	0.45	1.04
0.20	0.06	0.02	0.09	0.08	0.01	0.20	0.32	0.20	0.28	0.67
0.25	0.07	0.05	0.09	0.08	0.01	0.14	0.21	0.12	0.18	0.46
0.30	0.06	0.06	0.07	0.07	0.01	0.11	0.09	0.07	0.12	0.32
0.35	0.05	0.06	0.05	0.05	0.01	0.08	0.06	0.05	0.08	0.22
0.40	0.04	0.05	0.04	0.04	0.00	0.06	0.04	0.03	0.05	0.16
0.45	0.03	0.04	0.02	0.03	0.00	0.05	0.00	0.03	0.03	0.11
0.50	0.02	0.03	0.01	0.02	0.00	0.03	0.01	0.02	0.02	0.07
0.55	0.01	0.02	0.01	0.01	0.00	0.03	0.01	0.00	0.01	0.05
0.60	0.01	0.02	0.00	0.00	0.00	0.01	0.01	0.01	0.01	0.03
0.65	0.00	0.01	0.00	0.00	0.00	0.01	0.02	0.00	0.00	0.02
0.70	0.00	0.01	0.00	0.00	0.00	0.00	0.02	0.00	0.00	0.01
0.75	0.00	0.00	0.00	0.00	0.00	0.01	0.01	0.00	0.00	0.00
0.80	0.00	0.00	0.00	0.00	0.00	0.00	0.02	0.00	0.00	0.00
0.85	0.00	0.00	0.00	0.00	0.00	0.00	0.01	0.00	0.00	0.00
0.90	0.00	0.00	0.00	0.00	0.00	0.00	0.01	0.00	0.00	0.00
0.95	0.00	0.00	0.00	0.00	0.00	0.00	0.00	0.00	0.00	0.00
1.00	0.00	0.00	0.00	0.00	0.00	0.00	0.00	0.00	0.00	0.00

Table S14: Error in $\hat{C}$ when sampling by links.

q	Erdrey	Pref	Smallw	Renga	C. elegans	Airlines	Karate	Dolphins	Condmat	Power
0.05	0.51	0.20	0.00	0.01	0.05	0.15	–	–	0.02	0.05
0.10	0.36	0.05	0.00	0.01	0.04	0.05	–	–	0.00	0.27
0.15	0.21	0.00	0.00	0.00	0.00	0.06	–	–	0.01	0.03
0.20	0.20	0.02	0.00	0.00	0.02	0.01	–	–	0.00	0.02
0.25	0.01	0.00	0.00	0.00	0.01	0.00	–	0.19	0.00	0.04
0.30	0.00	0.00	0.00	0.00	0.02	0.02	0.16	0.06	0.00	0.01
0.35	0.05	0.00	0.00	0.00	0.00	0.01	0.05	0.07	0.00	0.00
0.40	0.03	0.01	0.00	0.00	0.00	0.01	0.08	0.07	0.00	0.01
0.45	0.02	0.01	0.00	0.00	0.00	0.01	0.05	0.03	0.00	0.02
0.50	0.01	0.00	0.00	0.00	0.00	0.00	0.08	0.02	0.00	0.03
0.55	0.01	0.00	0.00	0.00	0.01	0.00	0.01	0.02	0.00	0.01
0.60	0.01	0.00	0.00	0.00	0.01	0.01	0.06	0.02	0.00	0.01
0.65	0.01	0.00	0.00	0.00	0.00	0.00	0.00	0.02	0.00	0.00
0.70	0.00	0.01	0.00	0.00	0.01	0.00	0.02	0.01	0.00	0.00
0.75	0.00	0.00	0.00	0.00	0.00	0.01	0.02	0.01	0.00	0.00
0.80	0.00	0.00	0.00	0.00	0.00	0.00	0.05	0.02	0.00	0.00
0.85	0.01	0.00	0.00	0.00	0.00	0.00	0.01	0.01	0.00	0.00
0.90	0.00	0.00	0.00	0.00	0.00	0.00	0.01	0.01	0.00	0.00
0.95	0.00	0.00	0.00	0.00	0.00	0.00	0.00	0.00	0.00	0.00
1.00	0.00	0.00	0.00	0.00	0.00	0.00	0.00	0.00	0.00	0.00

Table S15: Error in $\hat{k}_{\max}$ when sampling by links.

q	Erdrey	Pref	Smallw	Renga	C. elegans	Airlines	Karate	Dolphins	Condmat	Power
0.05	0.67	0.00	0.20	0.16	0.11	0.18	1.14	2.38	0.06	0.24
0.10	0.33	0.00	0.05	0.37	0.01	0.09	0.62	1.39	0.15	0.37
0.15	0.30	0.00	0.10	0.18	0.00	0.14	0.42	0.02	0.12	0.19
0.20	0.28	0.01	0.10	0.40	0.02	0.10	0.36	0.02	0.12	0.07
0.25	0.17	0.00	0.05	0.23	0.03	0.06	0.32	0.10	0.08	0.13
0.30	0.17	0.00	0.03	0.24	0.01	0.07	0.16	0.11	0.09	0.01
0.35	0.15	0.00	0.00	0.27	0.00	0.04	0.15	0.03	0.08	0.02
0.40	0.19	0.00	0.00	0.20	0.01	0.04	0.11	0.05	0.07	0.01
0.45	0.11	0.00	0.00	0.11	0.00	0.05	0.13	0.04	0.07	0.01
0.50	0.07	0.00	0.00	0.16	0.01	0.03	0.13	0.04	0.05	0.04
0.55	0.01	0.00	0.00	0.15	0.00	0.03	0.09	0.06	0.05	0.03
0.60	0.09	0.00	0.00	0.17	0.01	0.03	0.06	0.02	0.03	0.05
0.65	0.08	0.00	0.00	0.13	0.01	0.01	0.09	0.04	0.03	0.01
0.70	0.07	0.00	0.00	0.10	0.00	0.02	0.06	0.02	0.02	0.02
0.75	0.02	0.00	0.00	0.07	0.01	0.02	0.06	0.02	0.01	0.02
0.80	0.01	0.00	0.00	0.03	0.00	0.00	0.04	0.03	0.02	0.04
0.85	0.00	0.00	0.00	0.04	0.00	0.00	0.03	0.03	0.02	0.04
0.90	0.01	0.00	0.05	0.02	0.01	0.00	0.01	0.01	0.01	0.02
0.95	0.01	0.00	0.05	0.00	0.00	0.00	0.00	0.02	0.00	0.01
1.00	0.00	0.00	0.00	0.00	0.00	0.00	0.00	0.09	0.00	0.00

Table S16: Error in $\hat{N}$ when sampling by interactions on an Erdös-Rényi random graph.

q	I	II	III	IV	V	VI	VII
0.05	0.54	0.50	0.46	0.41	0.36	0.34	0.36
0.10	0.48	0.39	0.30	0.23	0.18	0.14	0.18
0.15	0.42	0.28	0.19	0.12	0.09	0.06	0.10
0.20	0.35	0.20	0.12	0.07	0.05	0.03	0.05
0.25	0.29	0.14	0.07	0.04	0.02	0.01	0.03
0.30	0.24	0.10	0.05	0.02	0.01	0.01	0.02
0.35	0.19	0.07	0.03	0.02	0.01	0.00	0.01
0.40	0.14	0.05	0.02	0.01	0.01	0.00	0.01
0.45	0.11	0.03	0.01	0.01	0.00	0.00	0.01
0.50	0.08	0.02	0.01	0.00	0.00	0.00	0.00
0.55	0.06	0.01	0.01	0.00	0.00	0.00	0.00
0.60	0.05	0.01	0.00	0.00	0.00	0.00	0.00
0.65	0.03	0.01	0.00	0.00	0.00	0.00	0.00
0.70	0.02	0.00	0.00	0.00	0.00	0.00	0.00
0.75	0.01	0.00	0.00	0.00	0.00	0.00	0.00
0.80	0.01	0.00	0.00	0.00	0.00	0.00	0.00
0.85	0.01	0.00	0.00	0.00	0.00	0.00	0.00
0.90	0.00	0.00	0.00	0.00	0.00	0.00	0.00
0.95	0.00	0.00	0.00	0.00	0.00	0.00	0.00
1.00	0.00	0.00	0.00	0.00	0.00	0.00	0.00

Table S17: Error in $\hat{N}$ when sampling by interactions from a Scale-free weighted network.

q	I	II	III	IV	V	VI	VII
0.05	0.36	0.36	0.36	0.36	0.36	0.36	0.36
0.10	0.18	0.18	0.18	0.18	0.18	0.18	0.18
0.15	0.10	0.10	0.10	0.10	0.10	0.10	0.10
0.20	0.05	0.05	0.05	0.05	0.05	0.05	0.05
0.25	0.03	0.03	0.03	0.03	0.03	0.03	0.03
0.30	0.02	0.02	0.02	0.02	0.02	0.02	0.02
0.35	0.01	0.01	0.01	0.01	0.01	0.01	0.01
0.40	0.01	0.01	0.01	0.01	0.01	0.01	0.01
0.45	0.01	0.01	0.01	0.01	0.01	0.01	0.01
0.50	0.00	0.00	0.00	0.00	0.00	0.00	0.00
0.55	0.00	0.00	0.00	0.00	0.00	0.00	0.00
0.60	0.00	0.00	0.00	0.00	0.00	0.00	0.00
0.65	0.00	0.00	0.00	0.00	0.00	0.00	0.00
0.70	0.00	0.00	0.00	0.00	0.00	0.00	0.00
0.75	0.00	0.00	0.00	0.00	0.00	0.00	0.00
0.80	0.00	0.00	0.00	0.00	0.00	0.00	0.00
0.85	0.00	0.00	0.00	0.00	0.00	0.00	0.00
0.90	0.00	0.00	0.00	0.00	0.00	0.00	0.00
0.95	0.00	0.00	0.00	0.00	0.00	0.00	0.00
1.00	0.00	0.00	0.00	0.00	0.00	0.00	0.00

Table S18: Error in $\hat{M}$ when sampling by interactions from an Erdös-Rényi weighted network.

q	I	II	III	IV	V	VI	VII
0.05	0.00	0.85	0.78	0.72	0.66	0.65	0.67
0.10	0.00	0.71	0.60	0.49	0.40	0.41	0.44
0.15	0.00	0.59	0.44	0.32	0.22	0.24	0.29
0.20	0.00	0.48	0.31	0.19	0.10	0.12	0.19
0.25	0.00	0.38	0.21	0.10	0.02	0.05	0.13
0.30	0.00	0.30	0.13	0.03	0.02	0.00	0.08
0.35	0.00	0.22	0.07	0.01	0.05	0.02	0.06
0.40	0.00	0.16	0.02	0.04	0.06	0.04	0.04
0.45	0.00	0.11	0.01	0.05	0.06	0.04	0.03
0.50	0.00	0.07	0.03	0.05	0.05	0.04	0.02
0.55	0.00	0.03	0.04	0.04	0.04	0.03	0.01
0.60	0.00	0.01	0.04	0.04	0.03	0.02	0.01
0.65	0.00	0.01	0.04	0.03	0.02	0.02	0.01
0.70	0.00	0.02	0.03	0.02	0.01	0.01	0.01
0.75	0.00	0.02	0.02	0.01	0.01	0.01	0.01
0.80	0.00	0.02	0.02	0.01	0.00	0.00	0.00
0.85	0.00	0.02	0.01	0.00	0.00	0.00	0.00
0.90	0.00	0.01	0.00	0.00	0.00	0.00	0.00
0.95	0.00	0.00	0.00	0.00	0.00	0.00	0.00
1.00	0.00	0.00	0.00	0.00	0.00	0.00	0.00

Table S19: Error in $\hat{M}$ when sampling by interactions Scale-free weighted network.

q	I	II	III	IV	V	VI	VII
0.05	0.67	0.67	0.67	0.67	0.67	0.67	0.67
0.10	0.44	0.44	0.44	0.44	0.44	0.44	0.44
0.15	0.29	0.29	0.29	0.29	0.29	0.29	0.29
0.20	0.19	0.19	0.19	0.19	0.19	0.19	0.19
0.25	0.13	0.13	0.13	0.13	0.13	0.13	0.13
0.30	0.08	0.08	0.08	0.08	0.08	0.08	0.08
0.35	0.06	0.06	0.06	0.06	0.06	0.06	0.06
0.40	0.04	0.04	0.04	0.04	0.04	0.04	0.04
0.45	0.03	0.03	0.03	0.03	0.03	0.03	0.03
0.50	0.02	0.02	0.02	0.02	0.02	0.02	0.02
0.55	0.01	0.01	0.01	0.01	0.01	0.01	0.01
0.60	0.01	0.01	0.01	0.01	0.01	0.01	0.01
0.65	0.01	0.01	0.01	0.01	0.01	0.01	0.01
0.70	0.01	0.01	0.01	0.01	0.01	0.01	0.01
0.75	0.01	0.01	0.01	0.01	0.01	0.01	0.01
0.80	0.00	0.00	0.00	0.00	0.00	0.00	0.00
0.85	0.00	0.00	0.00	0.00	0.00	0.00	0.00
0.90	0.00	0.00	0.00	0.00	0.00	0.00	0.00
0.95	0.00	0.00	0.00	0.00	0.00	0.00	0.00
1.00	0.00	0.00	0.00	0.00	0.00	0.00	0.00

Table S20: Error in $\hat{k}_{\text{avg}}$ when sampling by interactions from an Erdös-Rényi weighted network.

q	I	II	III	IV	V	VI	VII
0.05	0.35	0.90	0.85	0.80	0.75	0.74	0.76
0.10	0.33	0.79	0.69	0.59	0.49	0.48	0.53
0.15	0.29	0.68	0.53	0.40	0.28	0.28	0.35
0.20	0.26	0.57	0.38	0.24	0.14	0.14	0.23
0.25	0.23	0.46	0.26	0.13	0.04	0.06	0.15
0.30	0.19	0.36	0.17	0.05	0.01	0.01	0.10
0.35	0.16	0.27	0.10	0.00	0.04	0.02	0.07
0.40	0.13	0.20	0.04	0.03	0.05	0.03	0.05
0.45	0.10	0.14	0.01	0.04	0.05	0.04	0.03
0.50	0.08	0.09	0.02	0.05	0.04	0.04	0.02
0.55	0.06	0.05	0.03	0.04	0.03	0.03	0.02
0.60	0.04	0.02	0.04	0.04	0.03	0.02	0.01
0.65	0.03	0.00	0.03	0.03	0.02	0.02	0.01
0.70	0.02	0.01	0.03	0.02	0.01	0.01	0.01
0.75	0.01	0.02	0.02	0.01	0.00	0.01	0.01
0.80	0.01	0.02	0.01	0.01	0.00	0.00	0.00
0.85	0.01	0.01	0.01	0.00	0.00	0.00	0.00
0.90	0.00	0.01	0.00	0.00	0.00	0.00	0.00
0.95	0.00	0.00	0.00	0.00	0.00	0.00	0.00
1.00	0.00	0.00	0.00	0.00	0.00	0.00	0.00

Table S21: Error in $\hat{k}_{\text{avg}}$ when sampling by interactions from a Scale-free weighted network.

q	I	II	III	IV	V	VI	VII
0.05	0.76	0.76	0.76	0.76	0.76	0.76	0.76
0.10	0.53	0.53	0.53	0.53	0.53	0.53	0.53
0.15	0.35	0.35	0.35	0.35	0.35	0.35	0.35
0.20	0.23	0.23	0.23	0.23	0.23	0.23	0.23
0.25	0.15	0.15	0.15	0.15	0.15	0.15	0.15
0.30	0.10	0.10	0.10	0.10	0.10	0.10	0.10
0.35	0.07	0.07	0.07	0.07	0.07	0.07	0.07
0.40	0.05	0.05	0.05	0.05	0.05	0.05	0.05
0.45	0.03	0.03	0.03	0.03	0.03	0.03	0.03
0.50	0.02	0.02	0.02	0.02	0.02	0.02	0.02
0.55	0.02	0.02	0.02	0.02	0.02	0.02	0.02
0.60	0.01	0.01	0.01	0.01	0.01	0.01	0.01
0.65	0.01	0.01	0.01	0.01	0.01	0.01	0.01
0.70	0.01	0.01	0.01	0.01	0.01	0.01	0.01
0.75	0.01	0.01	0.01	0.01	0.01	0.01	0.01
0.80	0.00	0.00	0.00	0.00	0.00	0.00	0.00
0.85	0.00	0.00	0.00	0.00	0.00	0.00	0.00
0.90	0.00	0.00	0.00	0.00	0.00	0.00	0.00
0.95	0.00	0.00	0.00	0.00	0.00	0.00	0.00
1.00	0.00	0.00	0.00	0.00	0.00	0.00	0.00

Table S22: Error in $k_{\max}$ when sampling by interactions from an Erdös-Rényi weighted network.

q	I	II	III	IV	V	VI	VII
0.05	3.00	0.76	0.81	0.84	0.83	0.84	0.85
0.10	1.82	0.66	0.73	0.76	0.76	0.77	0.80
0.15	1.27	0.60	0.67	0.71	0.69	0.70	0.73
0.20	0.82	0.53	0.60	0.66	0.62	0.66	0.69
0.25	0.72	0.48	0.56	0.59	0.59	0.60	0.63
0.30	0.49	0.44	0.51	0.52	0.54	0.55	0.58
0.35	0.51	0.35	0.50	0.52	0.49	0.53	0.55
0.40	0.35	0.36	0.42	0.49	0.47	0.47	0.49
0.45	0.29	0.28	0.39	0.44	0.41	0.44	0.45
0.50	0.20	0.31	0.37	0.37	0.37	0.39	0.42
0.55	0.17	0.26	0.32	0.36	0.34	0.34	0.37
0.60	0.16	0.22	0.33	0.33	0.31	0.32	0.33
0.65	0.13	0.20	0.31	0.28	0.23	0.27	0.30
0.70	0.10	0.19	0.26	0.26	0.20	0.23	0.26
0.75	0.08	0.15	0.21	0.23	0.17	0.17	0.23
0.80	0.01	0.13	0.21	0.18	0.14	0.14	0.18
0.85	0.02	0.12	0.17	0.14	0.08	0.08	0.14
0.90	0.01	0.09	0.12	0.11	0.04	0.04	0.11
0.95	0.04	0.08	0.10	0.06	0.01	0.02	0.06
1.00	0.05	0.04	0.06	0.02	0.07	0.05	0.03

Table S23: Error in $k_{\max}$ when sampling by interactions from a Scale-free weighted network.

q	I	II	III	IV	V	VI	VII
0.05	0.85	0.85	0.85	0.85	0.85	0.85	0.85
0.10	0.80	0.80	0.80	0.80	0.80	0.80	0.80
0.15	0.73	0.73	0.73	0.73	0.73	0.73	0.73
0.20	0.69	0.69	0.69	0.69	0.69	0.69	0.69
0.25	0.63	0.63	0.63	0.63	0.63	0.63	0.63
0.30	0.58	0.58	0.58	0.58	0.58	0.58	0.58
0.35	0.55	0.55	0.55	0.55	0.55	0.55	0.55
0.40	0.49	0.49	0.49	0.49	0.49	0.49	0.49
0.45	0.45	0.45	0.45	0.45	0.45	0.45	0.45
0.50	0.42	0.42	0.42	0.42	0.42	0.42	0.42
0.55	0.37	0.37	0.37	0.37	0.37	0.37	0.37
0.60	0.33	0.33	0.33	0.33	0.33	0.33	0.33
0.65	0.30	0.30	0.30	0.30	0.30	0.30	0.30
0.70	0.26	0.26	0.26	0.26	0.26	0.26	0.26
0.75	0.23	0.23	0.23	0.23	0.23	0.23	0.23
0.80	0.18	0.18	0.18	0.18	0.18	0.18	0.18
0.85	0.14	0.14	0.14	0.14	0.14	0.14	0.14
0.90	0.11	0.11	0.11	0.11	0.11	0.11	0.11
0.95	0.06	0.06	0.06	0.06	0.06	0.06	0.06
1.00	0.03	0.03	0.03	0.03	0.03	0.03	0.03

Table S24: Number of messages from September 2008-November 2009. The number of "observed" messages in our database comprise a fraction of the total number of Twitter messages made during period of this study (September 2008 through November 2009). While our feed from the Twitter API remains fairly constant, the total # of tweets grows, thus reducing the % of all tweets observed in our database. We calculate the total # of messages as the difference between the last message id and the first message id that we observe for a given month. This provides a reasonable estimation of the number of tweets made per month as message ids were assigned (by Twitter) sequentially during the time period of this study. The % observed represent the percent of messages observed out of the estimated total. We also report the number observed messages that are replies to specific messages and the percentage of our observed messages which constitute replies.

Week	Start date	# Obsvd. Msgs. $\times 10^6$	# Total Msgs. $\times 10^6$	% Obsvd.	# Replies $\times 10^6$	% Replies
1	09.09.08	3.14	7.26	43.2	0.88	28.1
2	09.16.08	3.36	8.31	40.4	0.90	26.9
3	09.23.08	3.43	8.89	38.6	0.90	26.2
4	09.30.08	3.33	9.06	36.8	0.89	26.6
5	10.07.08	2.33	9.38	24.8	0.64	27.5
6	10.14.08	4.39	9.87	44.4	1.24	28.3
7	10.21.08	4.70	10.01	47.0	1.35	28.8
8	10.28.08	5.74	10.34	55.5	1.64	28.5
9	11.04.08	5.58	11.14	50.1	1.63	29.3
10	11.11.08	4.70	9.88	47.6	1.42	30.2
